# Germline variants in *CDKN2A* wild‐type melanoma prone families

**DOI:** 10.1002/1878-0261.70020

**Published:** 2025-03-12

**Authors:** Gjertrud T. Iversen, Marie Loeng, Amalie Lund Holth, Per E. Lønning, Jürgen Geisler, Stian Knappskog

**Affiliations:** ^1^ Department of Clinical Science, K.G. Jebsen Center for Genome‐Directed Cancer Therapy University of Bergen Bergen Norway; ^2^ Department of Oncology Haukeland University Hospital Bergen Norway; ^3^ Department of Oncology Akershus University Hospital Lørenskog Norway; ^4^ Institute of Clinical Medicine University of Oslo Norway

**Keywords:** cancer risk, germline variants, inheritance, melanoma

## Abstract

Germline pathogenic variants in *CDKN2A* are well established as an underlying cause of familial malignant melanoma. While pathogenic variants in other genes have also been linked to melanoma, most familial cases remain unexplained. We assessed pathogenic germline variants in 360 cancer‐related genes in 56 Norwegian melanoma‐prone families. The index cases were selected based on familial history of melanoma and/or multiple primary melanomas, along with previous negative tests for pathogenic *CDKN2A* variants. We found 6 out of 56 index individuals to carry germline pathogenic or likely pathogenic variants in *BRCA2*, *MRE11*, *ATM*, *MSH2*, *CHEK2*, and *AR*. One family member with melanoma (not index case) carried a pathogenic variant in *MAP3K6*. In addition, we found a high fraction of variants previously considered benign and/or as variants of uncertain significance in xeroderma pigmentosum‐related genes. In particular, *XPC* ^*L48F*^ was found in 8 indexes; thus, the allele fraction (0.07) was significantly higher than in comparable healthy populations (0.02–0.03; *P*‐values from 0.007 to 0.014). In conclusion, we found that several melanoma‐prone families have pathogenic variants in genes not usually linked to melanoma.

## Graphical abstract

Among melanoma‐prone families, wild‐type for *CDKN2A* and *CDK4*, some have pathogenic variants in genes not usually linked to melanoma. Furthermore, rare XP‐related variants and variants in *MC1R* are enriched in such families.

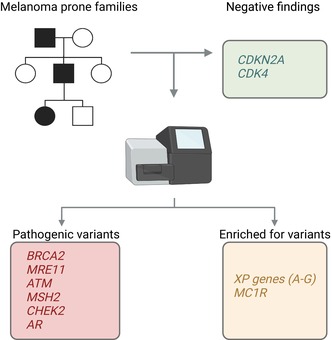

**Abbreviations**
AFA: adaptive focused acoustics
FFPE: formalin fixated paraffin embedded
MAF: minor allele frequency
MLPA: multiplex ligand probe amplification
PV: pathogenic variant
UV: ultraviolet (light)
VCF: variant call format
VUS: variant of uncertain significance
XP: *Xeroderma pigmentosum*

## 1 Introduction

While the risk of cutaneous malignant melanoma is strongly linked to ultraviolet radiation, about 5–10% occurs in individuals with a strong familial segregation of disease, pointing towards an inherited predisposition [1]. Around 25% of cases with a family history of melanoma carry germline pathogenic variants (PV) in the *CDKN2A* gene, encoding p16^INK4a^ and p14^ARF^ [2, 3] and for a similar fraction of cases, linkage to the *CDKN2A* locus is observed without detection of the underlying PV/genetic alteration.

A few melanoma‐prone families, with no linkage to *CDKN2A*, have been found to carry PVs in *CDK4* [3, 4, 5, 6], thus having alterations affecting the same cellular mechanism as *CDKN2A*, since p16^INK4a^ is a direct inhibitor of CDK4 function [7]. In addition, several other genes involved in different cellular processes have also been identified as melanoma susceptibility genes. This includes *BAP1*, encoding a BRCA1‐binding protein and thus involved in DNA repair [3, 8, 9], and *ATM*, involved in the general DNA damage response [10]. Further, several genes like *TERT*, *POT1*, *ACD*, and *TERF21P*, which all have functions related to telomere maintenance and stability, have been identified as melanoma susceptibility genes [3, 9, 11, 12]. Also, PVs have been found in *MITF*, a melanocyte lineage‐specific transcription factor, regulating the differentiation and proliferation of melanocytes [3, 9, 13]. However, the fraction of melanoma cases with underlying inherited predisposition by alterations in these genes is relatively low compared to *CDKN2A*, and in about 50% of familial cases of malignant melanoma, the underlying cause remains unexplained.

In a comprehensive study by Huang et al. [14], the prevalence of germline pathogenic and likely pathogenic variant mutations was summarized across 33 cancer types. The findings largely validated previous reports highlighting the importance of tumor suppressor genes such as *ATM*, *BRCA1*, *BRCA2*, and *PALB2* for a limited number of tumor types. Further, multi‐cancer risk genes such as *TP53*, *RB1*, and *MEN1* are associated with a defined, limited spectrum of cancers. However, the data also show that genes in general associated with specific cancer forms, in some cases, may be associated with different cancer types [4, 14]. In a recent study, this was also seen for melanoma, with pathogenic variants in genes involved in DNA repair, but not usually linked to melanoma, found in 10% of patients in a selected melanoma cohort [15]. Thus, it is possible that germline variants in a broader repertoire of genes may explain many familial melanomas, arguing for a broader screening for such variants in affected individuals and families.

In the present study, we assessed the germline status in *CDKN2A‐negative* individuals with a clear high melanoma‐risk profile, defined either by multiple primary melanomas in the index patient or by multiple family members with a melanoma diagnosis, across a targeted gene panel of 360 cancer‐related genes.

## 2 Materials and methods

### 2.1 Patients

The present study included 56 index individuals. Out of these, 53 were either previously treated for multiple primary melanomas or for a single melanoma but with several relatives with a melanoma diagnosis. Index individuals without a melanoma diagnosis themselves but with several relatives with melanoma were also allowed (*n* = 3). All types of primary melanomas were allowed (cutaneous melanoma, uveal melanomas, melanomas of the genitourinary tract etc.). We also included patients with metastatic melanoma of unknown origin when other family members had been diagnosed with melanoma as well. All patients were ≥ 18 years old and gave their written informed consent prior to participation. Relatives of all enrolled patients were invited to participate if aged > 18 years. All in all, 273 relatives were enrolled following extensive genetic counseling and written informed consent. Most index individuals had previously tested negative for pathogenic germline variants in *CDKN2A* in routine diagnostics but were centrally reanalyzed after inclusion in the present study. None were excluded; all 56 indexes were negative for *CDKN2A* pathogenic variants and included for further analyses. In a few cases, for validation of inheritance when blood samples were not available, we were able to recover melanoma tumor tissue samples from deceased relatives known to be diagnosed with a melanoma during their lifetime. For frequency comparison, we used available data from the Swedish cohort included in gnomAD (very similar to the Norwegian population; i.e., Scandinavian) and the European data from the 1000 Genomes Project [16].

### 2.2 DNA isolation

DNA from full blood was isolated using the QIAamp DNA Mini Kit (Qiagen, Hilden, Germany) according to the *DNA Purification from Blood or Body Fluids* spin protocol provided by the manufacturer, with the exception that 400 μL full blood was used as starting material. Elution was performed twice with 100 μL AE buffer, yielding a total volume of 200 μL purified DNA.

Individuals that were not able to deliver blood samples (study ID C06‐06, C06‐03, and A05‐40) donated saliva samples instead. DNA was isolated from saliva using the Oragene DNA OG‐600 kit (DNA genotek, Ottawa, ON, Canada). The procedure was performed according to the manufacturer's protocol for purification from 0.5 mL sample. In brief, the sample collection tube was incubated at 50 °C in a hot‐air incubator for 3 h, 0.5 mL sample was transferred to a new tube and mixed with 20 μL PT‐L2P reagent. Samples were incubated on ice for 10 min, followed by centrifugation of turbidity for 5 min at 15 000 ***g*.** The clear supernatant was mixed with 600 μL absolute ethanol in a new tube and incubated for 10 min for DNA precipitation. The DNA was pelleted by centrifugation for 2 min at 15 000 ***g*** followed by ethanol wash before dissolving the DNA pellet in 50 μL 10 mm Tris/HCl (0.5 mm EDTA, pH 9). Finally, for total rehydration, the DNA was left overnight in elution buffer at room temperature.

In several families, relatives of the index patients were deceased when the present study was conducted, and blood or saliva samples of these relatives were therefore not available. In some cases, formalin‐fixated paraffin‐embedded (FFPE) tumor specimens from these individuals were available. Tissue was collected from FFPE blocks either as sections of, on average, 20 μm thickness, or as cores of 1 mm size. DNA was isolated as previously described [17]. In brief, the Covaris adaptive focused acoustics (AFA) procedure was used, according to manufacturer's protocol, section C, with minor changes, applying a Covaris M220 focused‐ultrasonicator (Covaris, Woburn, MA, USA). Elution volume was 50 μL BE buffer (5 mm Tris/HCl, pH 8.5). Prior to further analyses, the isolated DNA was repaired from damages induced by the FFPE procedure, using the PreCR repair mix (New England Biolabs, Ipswich, MA, USA) and dNTP mixture (Takara, Kusatsu, Shiga, Japan) according to manufacturer's sequential reaction protocol.

### 2.3 Targeted cancer gene panel sequencing

DNA sequencing of a targeted 360‐cancer gene panel was carried out as previously described [18]. In brief, Illumina libraries were prepared applying the Kapa Hyper Prep kit (Kapa Biosystem, Wilmington, MA, USA) and the Agilent SureSelect XT kit (Agilent, Santa Clara, CA, USA). Targeted enrichment was performed using RNA baits (SureSelect, Agilent), targeted against a panel of 360 cancer‐related genes, ±5 bp of each exon to cover splice sites [18]. Libraries were sequenced on a MiSeq instrument (Illumina, San Diego, CA, USA) to an average mean depth of 231× for blood samples and 171× for FFPE samples (sequencing metrics in Table S1). A full list of the 360 genes is given in Table S2. Among the best‐described genes in which variants are specifically linked to melanoma, the 360 gene panel contained *CDKN2A*, *CDK4*, *BAP1*, *TERT*, and *MITF* (in addition to many more general cancer risk genes). Melanoma‐related genes that were not included were *POT1*, *TERF2D*, *ACD*, *TINF*, and *OCA2*.

### 2.4 Data analysis of targeted cancer gene panel sequencing

Targeted 360 cancer‐gene panel sequencing data were first analyzed (mapped to the human reference genome hg19 and variant calling) with the built‐in Miseq Reporter software on the Miseq instrument (Illumina) before the variant output files (vcf files) were annotated with ANNOVAR for gene context [19]. Post‐processing filters were applied to variant output data, resulting in a list of variants sharing the same properties. These were only exonic variants with a variant allele frequency of more than 0.1, while synonymous single nucleotide variants with less than 5 mutated reads and depth lower than 20× were excluded. All variants were subjected to population filtering using the data from 1000GenomesProject_2014okt_all, 1000GenomesProject_2014okt_ EUR and Esp6500siv2_all, to keep the variants with less than 3% minor allele frequency (MAF). All variants of interest were validated by manual inspection of mapping and read quality of sequence reads in IGV. In the main analysis, the resulting variants were grouped as either pathogenic, variants of uncertain significance (VUS) or benign based on literature review and assessment of information in publicly available databases, including Clinvar, Cosmic, PubMed, the Genome Aggregation Database, and databases specific for some genes of interest, such as BRCA Exchange. In a secondary, more stringent analysis, we performed pathogenicity assessment of variants, applying the Charger algorithm [20] as previously described [21].

### 2.5 *CDKN2A* deletion screening

In addition to *CDKN2A* variant assessment from the targeted sequencing data, small copy number variation in index patients was analyzed with Multiplex ligand probe amplification (MLPA) assay and coffalyser software (v. 140721.1958 and v. 220401.0000 MRC Holland, Amsterdam, The Netherlands). SALSA MLPA Probe kit P419‐A2, specific for *CDKN2A*, *CDKN2B*, and *CDK4*, was used (MRC Holland Amsterdam, The Netherlands) and the assay was performed according to the manufacturer's manual. As a positive control, we included DNA from a previous study where a deletion was known to remove the MLPA probe binding site in exon 1α [22].

Further, index cases were screened for a 10 kb deletion in *CDKN2A*, previously detected in a Norwegian melanoma‐prone family [22]. Analyses were performed by a PCR specific for the deletion's breakpoint, as previously described [22] (Fig. S1).

### 2.6 PCR amplification and sanger sequencing

Regions with variants of interest, detected in index cases, were analyzed in family members by PCR amplification and capillary sequencing. In addition, the *MC1R* gene was not covered by our 360 gene panel and, therefore, analyzed separately by PCR and capillary sequencing. The specific regions were amplified with specific primers and Taq Polymerase (Avantor, Radnor Township, PA, USA) according to the manufacturer's instructions. In general, amplification was carried out in the final concentrations in a mastermix of PCR‐clean water, 1x Key buffer with MgCl, 0.2 mm dNTPs, 0.2 μm forward and reverse primer (Table S3) and Taq polymerase. 1 μL gDNA was used as template; for low‐quality DNA from FFPE, 3–5 μL was used. PCR thermocycling started with denaturation at 95 °C for 5 min, followed by 35 cycles of 95 °C for 30 s, TM °C (see Table S3) for 30 s, and 72 °C for 30 s. A final elongation at 72 °C for 10 min was followed by cooling the samples to 10 °C. The correct size of the amplified product was confirmed by agarose gel electrophoresis along with Generuler DNA ladder mix (Thermo Scientific, Waltham, MA, USA). Amplified PCR products were cleaned up prior to sequencing reaction with Exozap Illustra Exoprostar 1‐step enzymatic PCR and sequencing reaction clean‐up kit (GE Healthcare Life Sciences, Buckinghamshire, UK). Amplification for capillary sequencing was conducted according to the manufacturer's recommendations with BigDye terminator version 1.1 (Applied Biosystems, Waltham, MA, USA) and appropriate primers in a 10 μL reaction volume. Thermocycling included denaturation at 94 °C for 5 min followed by 30 cycles of 94 °C for 15 s, 50 °C for 5 s, and 60 °C for 4 min. Finally, the samples were cooled to 10 °C. Capillary chromatograms were obtained by analyses on an automated DNA sequencer ABI 3730 DNA Analyzer.

### 2.7 Ethics approval and consent to participate

The study and its protocol were approved by the Regional Ethics Committee in the South‐Eastern Health Region of Norway (REK sør‐øst 2015/941). All participants provided written informed consent to genetic testing. All individuals with pathogenic or suspected pathogenic variants were referred to genetic counseling. Ethical approval was received (REK sør‐øst 27863‐2020) to recover archival tumor tissue from deceased relatives in a few families, if necessary, to strengthen the genetic findings of our study. The study methodologies conformed to the standards set by the Declaration of Helsinki.

## 3 Results

Index individuals with either a history of ≥ 2 primary melanomas or a recorded family history of melanoma (see Section 2) were included in the present study in order to identify underlying pathogenic germline variants potentially explaining the phenotype. In total, 56 index individuals, along with 273 family members, were included in the study. All indexes were evaluated for phenotype, followed by molecular analysis, while 183 of the family members were subjected to molecular analysis due to findings in the index individual in their respective families. Molecular analysis included massive parallel sequencing of a targeted 360 cancer gene panel (for all indexes) and/or Sanger sequencing of selected genes. General demographics for the indexes and the families are summarized in Table 1 and the 360 genes in our panel are listed in Table S2.

**Table 1:** Demographics of index individuals. DESMM, desmoplastic malignant melanoma; ME, melanoma of the eye; MM, malignant melanoma; NMM, nodular malignant melanoma; SSMM, superficial spreading malignant melanoma.

**Index patients**		
Age at diagnosis—mean (range)	58 (30–81)	
	***n***	**%**
Total	56	100.0
Females	26	46.4
Males	30	53.6
At least one SSMM	14	25.0
At least one NMM	12	21.4
At least one DESMM	1	1.8
Primary MM – not specified	42	75.0
At least one ME	0	0.0
One primary MM and at least one relative with MM	17	30.4
2 primary MM	19	33.9
3 primary MM	9	16.1
4 primary MM	4	7.1
5 or more primary MM	1	1.8
Distant metastatis from MM	18	32.1
Died of metastatic MM	8	14.3
At least one other cancer diagnosis^a^	6	10.7
**Relatives to indexpatients (with blood samples)**		
	***n***	**%**
Total	273	100.0
At least one MM‐diagnosis	36	13.2
≥ 2 primary MM	2	0.7
Distant metastatis from MM	10	3.7
At least one ME	2	0.7
Died of metastatic MM	10	3.7
At least one other cancer diagnosis^b^	25	9.2

a Ca. Coli: 1; Ca. Thyreoidea: 2; Ca. Pulm.: 1; Kidney Ca.: 1; Ca. Duodeni: 1.

b Breast‐ca: 7; Ca. Coli: 5; Ca. Thyreoidea: 3; Ca. Prost: 3; Ca.Endometr.: 2; Ca. Pancreas: 1; Kidney Ca.: 1; Ca. Pulm: 1; Ca. Uteri: 1; Ca. Duodenum: 1.

### 3.1 Status *CDKN2A* , *CDK4* and known melanoma risk genes

Most of the index individuals included in the study were previously tested and found negative for germline pathogenic variants (PV) in *CDKN2A* and *CKDK4* as part of routine diagnostics at their respective local hospitals. We conducted centralized re‐analysis of these genes in all index individuals by extracting data from targeted sequencing of a 360‐cancer gene panel and found no PVs in *CDKN2A* nor *CDK4*, confirming the previous routine testing results.

In a previous analysis of a Norwegian melanoma‐prone family, we identified a large 10 kb deletion removing *CDKN2A* exon 1α and half of exon 2 (breakpoint inside exon 2) [22]. Assessing all index individuals in the present study by a PCR specific for the breakpoint, all were found negative for the 10 kb deletion (Fig. S1B). Further, no deletions in *CDKN2A*, *CDKN2B*, *or CDK4* were observed in MLPA analysis.

Among other established melanoma‐related genes that were included in our 360 gene panel and found negative in all index individuals were *BAP1*, *TERT*, and *MITF*.

### 3.2 Pathogenic germline variants

**Fig. 1:**
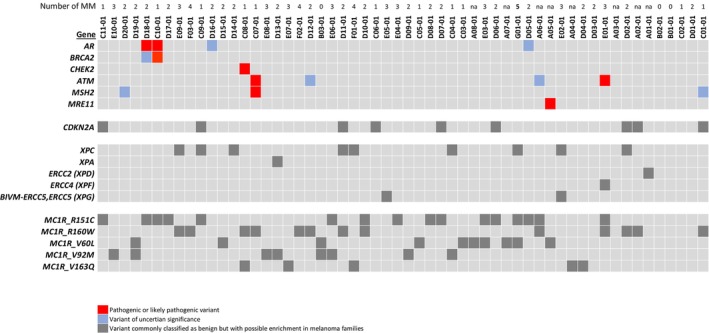
Germline variants in melanoma‐prone families. Oncoplot presenting the germline variants detected in selected genes (rows) in index patients (columns) of melanoma‐prone families. Red squares indicate pathogenic or likely pathogenic variants, blue squares indicate variants of uncertain significance (VUS) while dark gray squares indicate variants previously defined as benign but with a possible enrichment in the present cohort. Numbers on top indicate the number of primary malignant melanomas in each index individual (out of 56 enrolled indexes, 53 had one or more melanoma diagnoses, while 3 were included on the basis of family history alone). Out of the 6 indexes with pathogenic variants, two had more than one melanoma, three had a single melanoma, and one had an unknown number of melanomas.

In total, within the panel of 360 analyzed cancer genes, we found that 6 out of 56 index individuals carried pathogenic or likely pathogenic germline variants (Fig. 1). In addition, we found that most indexes carried variants of uncertain significance (VUS) and/or variants that have been previously classified as benign but that were enriched in our cohort (Fig. S2).

### 3.3 BRCA2

**Fig. 2:**
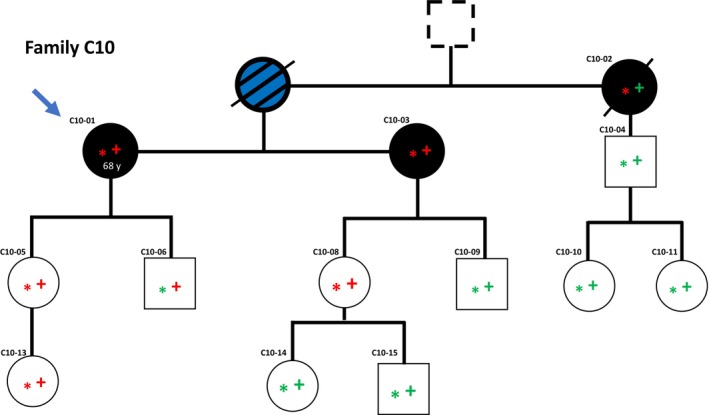
Family pedigree and germline variant overview for family C10. Blue arrow indicates the index individual. Asterisk (*) indicates individual status for the *BRCA2:NM_000059:exon11:c.G5857T:p.E1953X* variant; red asterisk indicates variant carriers while green asterisk indicates wild‐type allele only. A plus symbol (+) indicates individual status for the *AR:NM_000044:exon6:c.C2395G:p.Q799E* variant; red + indicates variant carriers while green + indicates wild‐type allele only. Black color indicates a diagnosis of malignant melanoma while white color indicates no melanoma diagnosis. Diagonal line indicates deceased individuals. Black and blue fill indicates malignant melanoma and additional cancer diagnosis (pancreatic cancer). Dashed outer line indicates that malignant melanoma is suspected based on self‐reporting from family members but has not been confirmed by medical records.

Sequencing of the index patient in family C10 (C10‐01), revealed a single nucleotide substitution from G to T at position 5857 in the *BRCA2* gene. This results in a premature stop in exon 11, generating a known pathogenic variant (*BRCA2* ^E1953X^) that has been linked to breast and ovarian cancer [23]. The variant co‐segregated with malignant melanoma in family C10, strongly indicating melanoma pathogenicity. No case of the more expected cancers (breast or ovarian cancer) was recorded among the individuals harboring the variant (Fig. 2). In addition to the index patient, a sister diagnosed with 4 primary melanomas, and an aunt with melanoma both carried the *BRCA2* ^E1953X^ variant. Samples from the index' mother were not available but based on inheritance and the finding in samples from the aunt, the mother, who suffered from both melanoma and pancreatic cancer, must have been a *BRCA2* ^E1953X^ carrier.

A second index patient (D18‐01; family D18) was also found to carry a rare *BRCA2* variant (*BRCA2* ^H114R^). This variant has previously been reported to be of uncertain significance and did not segregate with melanoma diagnoses in the family (Fig. S3).

### 3.4 *MRE11* and *MAP3K6*

**Fig. 3:**
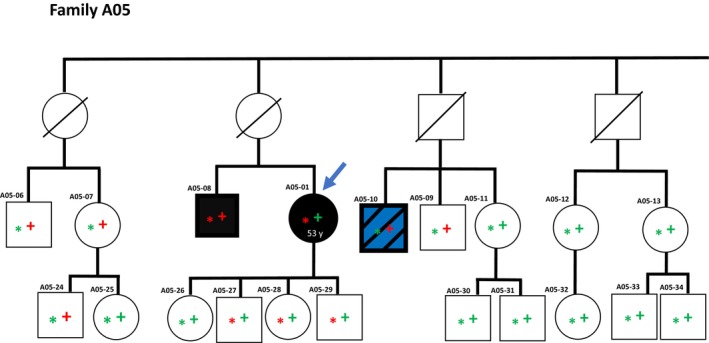
Family pedigree with an overview of gene variants in family A05. The blue arrow indicates the index individual. An asterisk (*) indicates individual status for the MRE11:NM_005591:exon4:c.201delC:p.S68Qfs*12 variant; a red asterisk indicates variant carriers, while a green asterisk indicates wild‐type allele only. A plus symbol (+) indicates individual status for the MAP3K6:NM_004672:exon19:c.2544delC:p.F849Sfs*143 variant; a red + indicates variant carriers, while a green + indicates wild‐type allele only. Black color indicates a diagnosis of malignant melanoma, while white color indicates no melanoma diagnosis. A diagonal line indicates deceased individuals. Black and blue fill indicates malignant melanoma and additional cancer diagnosis (rectal cancer).

The index patient in family A05 (A05‐01), diagnosed with skin melanoma, had a single nucleotide deletion early in the *MRE11* gene resulting in a frameshift and premature stop codon (*MRE11* ^S68Qfs*12^). This variant has not been reported previously, but given the early truncation, we considered this to be a pathogenic variant. Supporting pathogenicity in family A05, the *MRE11* ^S68Qfs*12^ variant co‐segregated with melanoma (Fig. 3).

Notably, a cousin of the index (A05‐10) who was diagnosed with ocular melanoma and a rectal cancer, did not carry the *MRE11* ^S68Qfs*12^ variant. However, this cousin was found to carry a single nucleotide frameshift deletion in *MAP3K6*, translated into an altered amino acid sequence and a premature stop codon (*MAP3K6* ^F849Sfs*143^). This variant has previously been classified as likely pathogenic [24]. While this variant was not found in the index patient, the index' brother (A05‐08), diagnosed with uveal melanoma, carried the *MAP3K6* ^F849Sfs*143^ variant in addition to the *MRE11* ^S68Qfs*12^ variant described above (Fig. 3).

### 3.5 *ATM* and *MSH2*

**Fig. 4:**
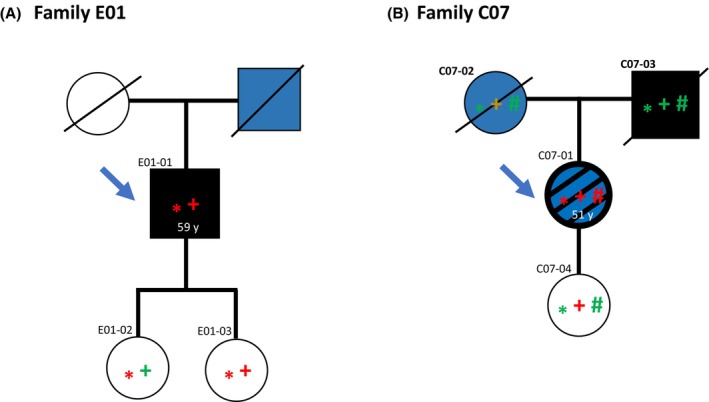
Family pedigree for family E01 and C07. Blue arrow indicates the index individual. (A) Family E01: Asterisk (*) indicates individual status for the ATM:NM_000051:exon22:c.3244_3247insTGdelC:p.H1082Lfs*13 variant; red asterisk indicates variant carriers while green asterisk indicates wild‐type allele only. A plus symbol (+) indicates individual status for the *ERCC4:NM_005236:exon11:c.A2546T:p.Q849L variant*; red + indicates variant carriers while green + indicates wild‐type allele only. Black fill indicates a diagnosis of malignant melanoma while white color indicates no melanoma diagnosis. Diagonal line indicates deceased individuals. Blue fill indicates cancer diagnosis other than melanoma (prostate cancer). (B) Family C07: Asterisk (*) indicates individual status for the *MSH2:NM_000251:exon12:c.1786_1788del:p.596_596del variant*; red asterisk indicates variant carriers while green asterisk indicates wild‐type allele only. A plus symbol (+) indicates individual status for the *TERT:NM_198253:exon2:c.1323_1325del:p.441_442del* variant; red + indicates variant carriers while green + indicates wild‐type allele only. Note that due to low‐quality samples, the sequencing of this deletion was not conclusive in C07‐02 and 03. A hash symbol (#) indicates individual status for the ATM truncating variant, *ATM:NM_000051:exon13:c.C1931A:p.S644X* variant; red # indicates variant carriers while green # indicates wild‐type allele only. The pathogenic variant in *MSH2* and *ATM* was not detected in any parent of the index, indicating these to be *de novo* variants. Black color indicates a diagnosis of malignant melanoma while white color indicates no melanoma diagnosis. Diagonal line indicates deceased individuals. Black and blue fill indicates malignant melanoma and additional cancer diagnoses (uterine cancer and basal cell carcinoma). Blue fill only indicates cancer diagnoses other than melanoma (breast, colon, and lung cancer).

In the index patient in family E01 (E01‐01), who had been treated for three primary melanomas, we found a complex insTGdelC variant in ATM resulting in a frameshift from H1082 and a premature termination of the protein after 13 amino acids. Judged by the premature stop codon and truncating effect on the protein by the H1082fs*13, this variant was defined as likely pathogenic (Fig. 4A).

In a second family (C07), the index patient (C07‐01) revealed a C to A nucleotide substitution at position 1931 in *ATM*. This variant (*ATM* ^S644X^), leading to a premature stop codon in exon 13, has been previously classified as pathogenic. Notably, in addition to the pathogenic *ATM* variant, C07‐01 also carried a known pathogenic variant in the DNA mismatch repair gene *MSH2*. This was a three‐nucleotide deletion at coding position 1786–1788 that translates to the deletion of Asparagine 596. Notably, neither the *ATM* nor the *MSH2* variant was detected in any of the index patient's parents, indicating that both variants may have occurred *de novo* (Fig. 4B).

### 3.6 CHEK2

**Fig. 5:**
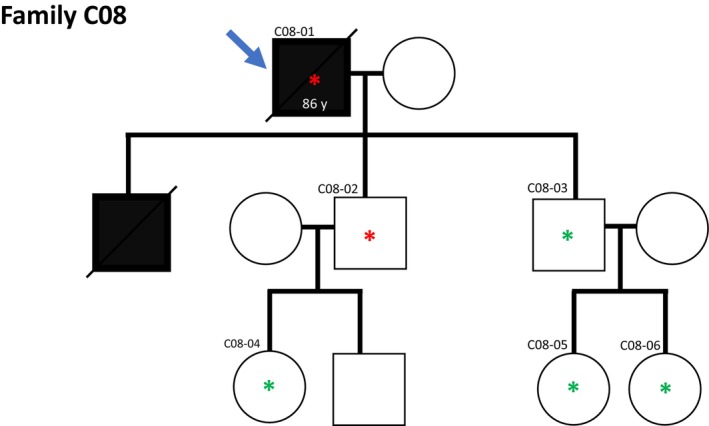
Family pedigree for family C08. Blue arrow indicates the index individual. Asterisk (*) indicates individual status for the *CHEK2:NM_007194:exon4:c.T470C:p.I157T* variant; red asterisk indicates variant carriers while green asterisk indicates wild‐type allele only. Black color indicates a diagnosis of malignant melanoma, while white color indicates no melanoma diagnosis. Diagonal line indicates deceased individuals.

The index patient of family C08 (C08‐01) carried a likely pathogenic variant in *CHEK2* (*CHEK2* ^I157T^). Among three sons of the index patient, two did not have a cancer diagnosis, one of whom was *CHEK2* wild‐type while the other carried the variant. The third son was diagnosed with metastatic malignant melanoma, but DNA for molecular analyses was not available (Fig. 5).

### 3.7 AR

Interestingly, in two families (D18 and C10), we found the same pathogenic variant in the androgen receptor gene, *AR* ^*Q799E*^ (Figs 1 and 2, Fig. S3). To the best of our knowledge, the two families were not related. In family D18, in addition to the index patient (D18‐01), *AR* ^*Q799E*^ was found in a healthy daughter and two grandchildren but not in another of the index' daughters (D18‐02) who had been diagnosed with malignant melanoma. In family C10, the index patient (C10‐01) and her sister (with 4 primary melanomas) both had the *AR* ^*Q799E*^ variant, but an aunt of the index patient, diagnosed with melanoma was found wild‐type for *AR* ^*Q799E*^. In addition to this lack of co‐segregation, notably, in both these two families we also detected *BRCA2* variants (a pathogenic variant in family C10 and a VUS in family D18; see above). Taken together, even if the *AR* ^*Q799E*^ is classified as pathogenic, it is not likely to be the direct underlying cause of melanoma.

### 3.8 Variants of uncertain significance and benign variants in melanoma‐related genes

In addition to the pathogenic and likely pathogenic variants, we found a large number of variants previously classified as variants of uncertain significance (VUS) or benign. Although the interpretation of such variants should be done with caution, the presence of some of these variants in our highly selected cohort was of particular interest due to segregation with melanoma and/or the general link between the genes in which the variants reside and melanoma.

In *CDKN2A*, the known A148T variant (rs3731249) was detected in 9 of 56 (16%) index individuals. While this variant is classified as benign by multiple sources, the observed frequency in our cohort was higher than the recorded frequency in healthy Europeans in the 1000 Genomes Project [16] (24 of 503; 4.8%; *P* = 0.003) and the closest population in gnomAD [25] (Swedish population; 942 of 13 057; 7.2%; *P* = 0.01).

The Neurofibromin 1 (*NF1*) gene is frequently somatically mutated in melanoma, and pathogenic germline variants in this gene are a known risk factor for melanoma, in addition to neurofibromatosis [26]. In the index patient of family D14 (D14‐01), we found a single nucleotide substitution in *NF1*, the D176E. This variant is classified as likely benign in the ClinVar database, but in addition to the index, the variant was also found in a second family member diagnosed with melanoma (D14‐05; Fig. S4).

The index patient in family A06 (A06‐01) carried variants in several classical tumor suppressor genes, including two different variants in *MLH1* (A441T and the K618A), a variant in *PALB2* (L939W) and several variants in *ATM* and *ATR*, all classified as VUS or benign. More interestingly, the index and her sister (A06‐02), both diagnosed with malignant melanoma, shared a double nucleotide substitution resulting in the *PTCH1* ^H739F^ variant. The *PTCH1* gene encodes the patched‐1 receptor protein, which prevents uncontrolled cell proliferation in early development [27], and germline variants have been linked to Gorlin syndrome (high risk of basal cell carcinoma); however, at present, the *PTCH1* ^H739F^ variant detected here is classified as a VUS or likely benign. The same variant was also seen in one healthy niece (age not known) of the index (A06‐04; daughter of A06‐02), while a second sister of the index (A06‐03) with uterine cancer was wild‐type for *PTCH1* (Fig. S5). Further, another *PTCH1* variant was found in family C11, where the index patient (C11‐01) carried *PTCH1* ^R665H^. This variant is currently classified as benign/likely benign, and it should be noted that it did not segregate with melanoma in family C11, since the brother of C11‐01 (C11‐03) was found to be *PTCH1* wild‐type while being diagnosed with melanoma (Fig. S6).

In the same family (C11), a variant in *JAK3* (*JAK3* ^V722I^) did segregate with melanoma. This variant has been reported as linked to AML, but there are also reports classifying it as VUS and as likely benign [28]. Adding further doubts to the impact of the *JAK3* ^V722I^ variant, it was also found in a second index patient (E09‐01) and in this family, it did not segregate with melanoma (Fig. S7).

### 3.9 Variants in xeroderma pigmentosum‐related genes

*Xeroderma pigmentosum* (XP) is a recessive condition characterized by UV sensitivity, lentigines, and early onset of skin cancer. It is mainly caused by variants in eight different genes: xeroderma pigmentosum complementation group A (*XPA*) through complementation group G (*XPG*) [29, 30]. In the present cohort, no individuals were diagnosed with XP. Among the 56 indexes, we found a total of 7 different variants in XP‐related genes (*XPA*, *XPC*, *XPD*, *XPF*, and *XPG*) in 13 individual index patients. All variants had VAFs indicating heterozygosity, but one patient harbored two variants. The most frequent single variant was *XPC* ^L48F^, which was detected in 8 out of 56 indexes (14.3%; minor allele frequency (MAF) of 0.071; Table S4). While the *XPC* ^L48F^ variant is classified as benign and we currently do not have strong data arguing for a reclassification, it should be noted that the frequency observed in our heavily selected cohort of high‐risk melanoma individuals was significantly higher than in comparable healthy populations (Swedish population in gnomAD: MAF = 0.028; *P* = 0.014 and European population in 1000 Genomes Project MAF = 0.022; *P* = 0.007). Regarding other variants, the *XPC* ^K481N^ variant was detected in a patient with 5 primary malignant melanomas (G01‐01) and a family history of widespread intestinal polyps, and another index patient (the one with two variants; E02‐01) carried an *XPG* ^A1036T^ variant in addition to the *XPC* ^L48F^ variant. We also detected variants of uncertain significance in other xeroderma pigmentosum‐associated genes, namely the *XPA* ^G6R^ (D13‐01), *XPF/ERCC4* ^Q849L^ (E01‐01), *XPG/ERCC5* ^E399K^ (E05‐01) and *XPD*/*ERCC2* ^P426L^ (A01‐01, Fig. 1, Table S4). Although the small number of observations precluded formal statistical assessments for these variants individually, it is noteworthy that the summarized allele fraction of XP‐related variants was 0.125 in our index individuals, contrasting with 0.032 in the European population of the 1000 Genomes Project (*P* = 7.1 × 10^−5^; Table S4; the corresponding fraction for gnomAD could not be calculated due to different total allele counts for the XP‐related variants).

### 3.10 Variants in *MC1R*

The melanocortin 1 receptor (*MC1R*) gene was analyzed in a separate gene‐specific analysis. Applying the same population frequency filters as for the NGS data, we detected 5 different *MC1R* variants in the index individuals (Fig. 1; Table S5). All these variants were more frequently detected in our cohort than in the cohorts of healthy individuals (Europeans in the 1000 Genome Project and the Swedish population in the gnomAD database) and for four of them, the difference was statistically significant towards at least one of the cohorts of healthy individuals (*P*‐values ranging from 0.009 to 0.1 for the individual comparisons; Table S5). Notably, the summarized allele fraction of *MC1R* variants (0.107) in our index individuals was also much higher than in the European population of the 1000 Genomes Project (0.021; *P* = 3.3 × 10^−5^; Table S5; the corresponding fraction for gnomAD could not be calculated due to different total allele counts for the *MC1R* variants).

In secondary analyses for *MC1R*, we also compared the allele frequencies of variants more common in the population. Here, we found the common Arg151Cys variant to be significantly enriched in our cohort (allele fraction 0.170 versus 0.072 and 0.077 in Europeans in the 1000 Genomes Project and the Swedish population in the gnomAD database, respectively; *P* = 0.001 for both comparisons; Fig. 1, Table S6). Notably, it has previously been reported that the combination of the *MC1R* ^Arg151Cys^ and *CDKN2A* ^A148T^ variants leads to an increased risk of melanoma. In our cohort, 4 of 19 indexes harboring the *MC1R* ^Arg151Cys^ overlapped with the 9 indexes harboring *CDKN2A* ^A148T^. As such, we did not see an enrichment of the combined genotype in our cohort (*P* > 0.4).

## 4 Discussion

In the majority of cases where inherited melanoma susceptibility variants are detected, the variants are linked to the *CDKN2A* gene. While PVs have also been detected in several other genes, including *CDK4* [3, 4, 5, 6], *BAP1* [3, 8, 9], *ATM* [10], *MITF* [3, 9, 13] and multiple telomere stability genes *TERT*, *POT1*, *ACD*, and *TERF21P* [3, 9, 11, 12], about half of the cases of familial melanoma remain unexplained.

The aim of the present work was to identify potential pathogenic germline genetic variants in melanoma‐prone families without any detected pathogenic alterations in *CDKN2A* or *CDK4*. While we did not identify any clear germline pathogenic variant in the majority of index patients, six index individuals were found to carry germline variants classified as pathogenic or likely pathogenic. The genes involved (*BRCA2*, *MRE11*, *ATM*, *MSH2*, *CHEK2*, and *AR*) are all implicated in different types of cancer, but pathogenic variants in any of these genes are not typically linked to malignant melanoma, perhaps with the exception of *ATM* [10]. As such, our present findings are in line with recent findings for melanoma [15] as well as an increasing realization that several cancer‐related genes may be implicated in several other cancer types than the one(s) originally linked to the gene [31]. It is also worth noting that in this set of families, we see a likely enrichment of other cancer forms in addition to malignant melanoma, which might indicate shared pathways for carcinogenesis.

Pathogenic germline variants in the breast cancer susceptibility gene, *BRCA2*, are commonly known for an increased risk of breast and/or ovarian cancer. Studies have also linked this gene to other types of cancer like pancreatic and prostate cancer [32] as well as uveal melanoma [33]. In our study, we found the *BRCA2* ^E1953X^ variant in the most frequently mutated exon 11. This variant is in the RAD51 binding area of BRCA2, important in DNA repair by homologous recombination [32, 34]. Interestingly, although seven individuals in family C10 affected by *BRCA2* ^E1953X^ were females and four of them had melanoma, none were diagnosed with breast or ovarian cancer. These results raise the question of whether there may be a yet unknown genetic factor present in this family skewing the cancer risk caused by the *BRCA2* variant towards melanoma rather than breast and ovarian cancer.

MRE11 is a nuclease involved in DNA double‐stranded break repair, constituting the MRN complex together with RAD50 and NBS1. Germline variants in *MRE11* have been detected in a few families with mesothelioma and breast cancer [35, 36]. In the present study, we found the *MRE11* ^S68Qfs*12^ variant in an index patient A05‐01 and one relative. The variant results in a premature stop codon at amino acid 80, meaning that most of the nuclease domain, the DNA binding domain, as well as both the RAD50 and NBN binding sites are lost.

ATM‐mediated phosphorylation of CHEK2 activates several downstream factors, resulting in the repair of DNA breaks or apoptosis [37]. In our cohort, we detected two pathogenic ATM variants, *ATM* ^S644X^ and *ATM* ^H1082fs*13^. The *ATM* ^S644X^ variant is previously reported to be associated with an increased risk of gastric [38], breast, and prostate cancer [39] while the *ATM* ^H1082fs*13^ variant has previously been described as a Norwegian founder mutation [40]. For both the detected variants, the premature stop codons result in the loss of the FAT and the protein kinase domain of ATM [37], indicating impaired function likely leading to an increased risk of cancer. None of these variants have previously been linked to malignant melanoma, but several other germline variants in *ATM* have been found in patients with multiple malignant melanomas [41] indicating a role for the gene in melanoma development.

Germline variants in *MSH2*, like *MLH1* and *MSH6*, are linked to Lynch syndrome characterized by increased risk for cancers in the colon, endometrium, and several other cancer forms [42]. To the best of our knowledge, germline mutations in *MSH2* have not previously been firmly linked to melanoma risk. We found a PV in *MSH2* in a patient also carrying a PV in ATM. Although it is likely that both variants have contributed to the disease, it is not possible, in our data, to assign a main risk effect to either of the two variants.

One family was found to harbor the *CHEK2* ^I157T^ variant. While the *CHEK2* gene is widely recognized as a multi‐organ cancer susceptibility gene, the role of *CHEK2* ^I157T^ has been debated, but several case–control analyses have shown a correlation between this variant and the susceptibility for various types of cancer, like breast, colorectal, and prostate cancer [43].

We found two index individuals to harbor the same variant in the *AR* gene. To the best of our knowledge, the two individuals were not related. The variant detected here (*AR* ^Q799E^) is linked to androgen insensitivity in males [44] and other variants in the gene have been linked to prostate cancer [45]. Notably, both the index cases in whom we detected the *AR* ^Q799E^ variant were females. While *AR* variants have not been linked directly to the risk of malignant melanoma, AR may play a role in melanoma treatment since it has been shown that AR blocking is mediating a better response to BRAF/MEK inhibition [46]. However, in both families where we detected the *AR* ^Q799E^ variant, it did not segregate with melanoma, and it is therefore unlikely that this variant is the underlying cause of the disease.

In addition to the variants in the indexes, we also detected a pathogenic *MAP3K6* variant in a melanoma‐diagnosed cousin in family A05. The MAPK signaling pathway is involved in several cellular processes, among others, proliferation and apoptosis. Germline variants in *MAP3K6* are often related to gastric cancer (24) while limited data are present for any link to melanoma. It should be noted, however, that the MAP3K6 protein is expressed at high levels in skin [47]. The *MAP3K6* ^F849Sfs*143^ variant detected in the present study is located in the protein kinase domain and results in a loss of a large portion of the protein [24]. Notably, the patient harboring this variant was not only diagnosed with a melanoma but also with rectal cancer.

Although these variants are classified as pathogenic and detected in highly melanoma‐prone individuals and families, corroboration in other families and/or extended linkage analyses may be necessary to firmly establish that melanoma risk is directly attributed to these variants.

All the variants we detected in XP‐related genes have previously been classified as VUS or benign. In the present study, we have no firm data arguing that any single one of these variants should be reclassified. Our study was not designed as a case–control study, and comparisons to other (but similar) populations should be interpreted with care. Still, it is interesting to note the significant enrichment of the *XPC* ^L48F^ variant in our cohort. Further, it is notable that the sum of different XP variants is also enriched. This finding may warrant assessments of XP‐related variants (individually or perhaps particularly, combined) in future larger case–control studies.

For the majority of included families, the underlying causes of melanoma remain unknown. Even if we did not detect any family harboring a specific large deletion in *CDKN2A*, previously found in Norway [22], it may be that other deletions or structural rearrangements could be an underlying cause in some of the families. Further, some index cases were included based on a history of multiple primary melanoma diagnoses, without any family history. Such cases may carry mosaic genetic variants not detected in the blood. Notably, as an alternative to mosaic pathogenic *genetic* variants, we have also recently found constitutional mosaic promoter methylation of *BRCA1* to be associated with a significantly increased risk of triple‐negative breast cancer and high‐grade serous ovarian cancer [48, 49]. Some individuals may have early‐life epigenetic alterations in tumor suppressor genes, conferring a high risk of melanoma in adult life. Further, non‐coding RNAs have also previously been linked to melanoma and in particular to multiple primary melanoma pathogenesis [50, 51], and it may be that such mechanisms could explain some of the unresolved cases in the present cohort.

## 5 Conclusions

We found that several melanoma‐prone families had pathogenic variants in genes not usually linked to melanoma. Furthermore, our data may indicate that rare XP‐related variants that are currently classified as benign could be linked to melanoma, pending further validation.

## Conflict of interest

*Research Funding (to Institution)*: AstraZeneca (SK, PEL), Novartis (PEL), Pfizer (SK, PEL). *Honoraria*: AstraZeneca (SK, PEL), Abbvie (PEL), Bristol‐Myers Squibb (JG), Dagens Medisin (PEL), Eli Lilly (JG), MSD (JG), Novartis (JG, SK), Pfizer (SK), Pierre Fabre (JG, SK, PEL), Roche (PEL). *Consulting or Advisory Role*: AstraZeneca (JG, PEL), Eli Lilly (JG), Laboratorios Farmacéuticos Rovi (PEL), MSD (JG), Novartis (JG). *Travel, Accommodations, Expenses*: Pierre Fabre (PEL). *Speakers' Bureau*: Akademikonferens (PEL), Aptitude Health (PEL), AstraZeneca (JG), Bristol‐Myers‐Squibb (JG), MSD (JG), Novartis (JG), Pfizer (JG), Pierre Fabre (JG). *Patents, Royalties, Other Intellectual Property*: Patent EP2389450 A1 (SK), Patent. WO 2012/010661 (SK), Cytovation (PEL). All remaining authors have declared no conflicts of interest.

## Author contributions

GTI: Methodology, Formal analysis, Investigation, Data curation, Writing – original draft. ML: Investigation, Data curation. ALH: Investigation, Data curation. PEL: Conceptualization, Writing – review & editing, Supervision. JG: Conceptualization, Data curation, Writing – review & editing, Supervision, Project administration. SK: Conceptualization, Formal analysis, Data curation, Writing – original draft, Supervision, Project administration.

### Peer review

The peer review history for this article is available at https://www.webofscience.com/api/gateway/wos/peer‐review/10.1002/1878‐0261.70020.

## Supporting information


**Fig. S1.** Analysis of intragenic *CDKN2A* deletion. (A) Schematic illustration of a > 13 000 bp deletion previously detected in a Norwegian melanoma‐prone family (ref). Deletion breakpoints are located in the intron between exons 1β and 1α and within exon 2, resulting in a truncated mRNA and a truncated p14^ARF^ protein as well as lack of expression of p16^INK4a^. PCR primers for detection of the deletion in genomic DNA are indicated as gray arrows. (B) Agarose gel images revealing negative results for the *CDKN2A* deletion (described above in A) for all index cases in the present study. The visible bands in the index samples are identified as primer dimers and single primers. Positive bands for the deletion are visible in the positive controls (“+”). Positive control was DNA from an affected individual (the index patient “XI”) from the previous report (8). Negative controls were mastermix without template (−) and sample previously analyzed and found to be negative for the deletion (n.s).

**Fig. S2.** Germline variants in melanoma‐prone families. Extended oncoplot presenting all germline variants detected in index patients of melanoma‐prone families. Red squares indicate pathogenic or likely pathogenic variants, blue squares indicate variants of uncertain significance (VUS) while dark gray squares indicate variants previously defined as benign but with a possible enrichment in the present cohort. Numbers on top indicate the number of primary malignant melanomas in each index individual.

**Fig. S3.** Family pedigree for family D18. Blue arrow indicates the index individual. Asterisk (*) indicates individual status for the *BRCA2:NM_000059:exon4:c.A341G:p.H114R* variant; red asterisk indicates variant carriers while green asterisk indicates wild‐type allele only. A plus symbol (+) indicates individual status for the *AR:NM_000044:exon6:c.C2395G:p.Q799E* variant; red + indicates variant carriers while green + indicates wild‐type allele only. Black color indicates a diagnosis of malignant melanoma while white color indicates no melanoma diagnosis. Diagonal line indicates deceased individuals.

**Fig. S4.** Family pedigree for family D14. Blue arrow indicates the index individual. Asterisk (*) indicates individual status for the *ERBB4:NM_005235:exon12:c.G1451A:p.R484K* variant; red asterisk indicates variant carriers while green asterisk indicates wild‐type allele only. A plus symbol (+) indicates individual status for the *XPC:NM_004628:exon2:c.C142T:p.L48F* variant; red + indicates variant carriers while green + indicates wild‐type allele only. A hash symbol (#) indicates individual status for the *NF1:NM_000267:exon5:c.T528A:p.D176E* variant; red # indicates variant carriers while green # indicates wild‐type allele only. Black color indicates a diagnosis of malignant melanoma while white color indicates no melanoma diagnosis. Diagonal line indicates deceased individuals.

**Fig. S5.** Family pedigree for family A06. Blue arrow indicates the index individual. Asterisk (*) indicates individual status for the *MLH1: NM_000249:exon12:c.G1321A:p.A441* variant; red asterisk indicates variant carriers while green asterisk indicates wild‐type allele only. A plus symbol (+) indicates individual status for a second *MLH1 variant, NM_000249:exon16:c.AA1852‐1853GC: p.K618A* variant; red + indicates variant carriers while green + indicates wild‐type allele only. A hash symbol (#) indicates individual status for the *PTCH1: NM_000264: exon14:c.CA2215TT:p.H739F* variant; red # indicates variant carriers while green # indicates wild‐type allele only. A double cross (‡) indicates individual status for the *PALB2:NM_024675:exon8:c.T2816G:p.L939W* variant; red indicates variant carriers while green indicates wild‐type allele only. Black color indicates a diagnosis of malignant melanoma while white color indicates no melanoma diagnosis. Blue color indicates cancer diagnosis other than melanoma; here stomach cancer (mother of index) and uterine cancer (sister of index).

**Fig. S6.** Family pedigree for family C11. Blue arrow indicates the index individual. Asterisk (*) indicates individual status for the *PTCH1:NM_000264:exon14:c.G1994A:p.R665H* variant; red asterisk indicates variant carriers while green asterisk indicates wild‐type allele only. A plus symbol (+) indicates individual status for the *JAK3:NM_000215:exon16:c.G2164A:p.V722I* variant; red + indicates variant carriers while green + indicates wild‐type allele only. Black color indicates a diagnosis of malignant melanoma while white color indicates no melanoma diagnosis. Blue color indicates cancer diagnosis other than melanoma (brain tumor). Diagonal line indicates deceased individuals. Yellow plus symbol indicates that the variant status is unknown due to restricted biomaterial and consequently, technical failure in analysis.

**Fig. S7.** Family pedigree for family E09. Blue arrow indicates the index individual. Asterisk (*) indicates individual status for the *XPC:NM_004628:exon2:c.C142T:p.L48F* variant; red asterisk indicates variant carriers while green asterisk indicates wild‐type allele only. A plus symbol (+) indicates individual status for the *JAK3:NM_000215:exon16:c.G2164A:p.V722I* variant; red + indicates variant carriers while green + indicates wild‐type allele only. Black color indicates a diagnosis of malignant melanoma while white color indicates no melanoma diagnosis.

**Table S1.** Sequencing metrics.
**Table S2.** Gene sequencing panel.
**Table S3.** Primers and annealing temperatures for PCRs.
**Table S4.** Allele frequencies of variants in XP‐related genes.
**Table S5.** Allele frequencies of selected *MC1R* variants.
**Table S6.** Allele frequencies of all *MC1R* variants.

**Data S1.** Materials and methods.

## Data Availability

The data generated in this study are not publicly available due to the germline genetic information that could compromise patient privacy but are available upon reasonable request from the corresponding author.
