# Supplementary figures and images for: Germline variants in CDKN2A wild‐type melanoma prone families
Source: Mol Oncol. 2025 Mar 12;19(5):1493–507. doi: 10.1002/1878-0261.70020 (PMC12077288; doi:10.1002/1878-0261.70020)

# Supplementary figure S1

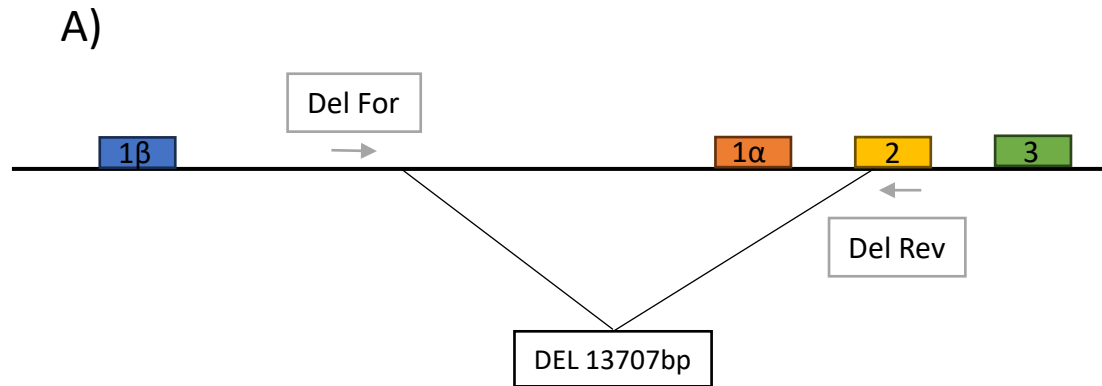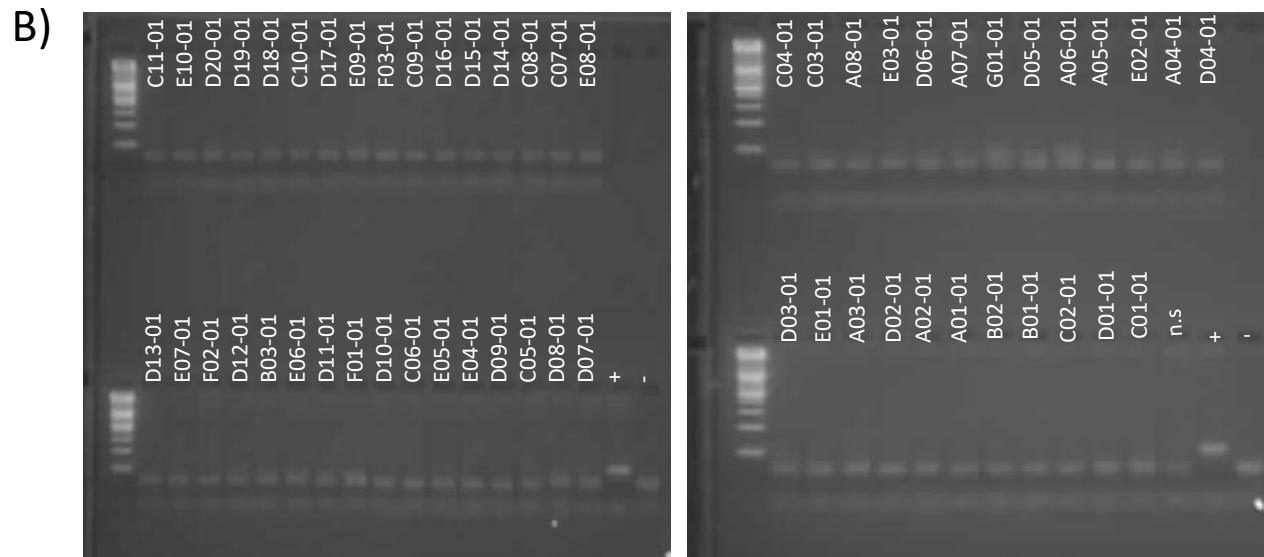

Supplement: Supplementary file 1 — Fig. S1. Analysis of intragenic CDKN2A deletion. (A) Schematic illustration of a > 13 000 bp deletion previously detected in a Norwegian melanoma‐prone family (ref). Deletion breakpoints are located in the intron between exons 1β and 1α and within exon 2, resulting in a truncated mRNA and a truncated p14ARF protein as well as lack of expression of p16INK4a. PCR primers for detection of the deletion in genomic DNA are indicated as gray arrows. (B) Agarose gel images revealing negative results for the CDKN2A deletion (described above in A) for all index cases in the present study. The visible bands in the index samples are identified as primer dimers and single primers. Positive bands for the deletion are visible in the positive controls (“+”). Positive control was DNA from an affected individual (the index patient “XI”) from the previous report (8). Negative controls were mastermix without template (−) and sample previously analyzed and found to be negative for the deletion (n.s). [file MOL2-19-1493-s004.pdf]

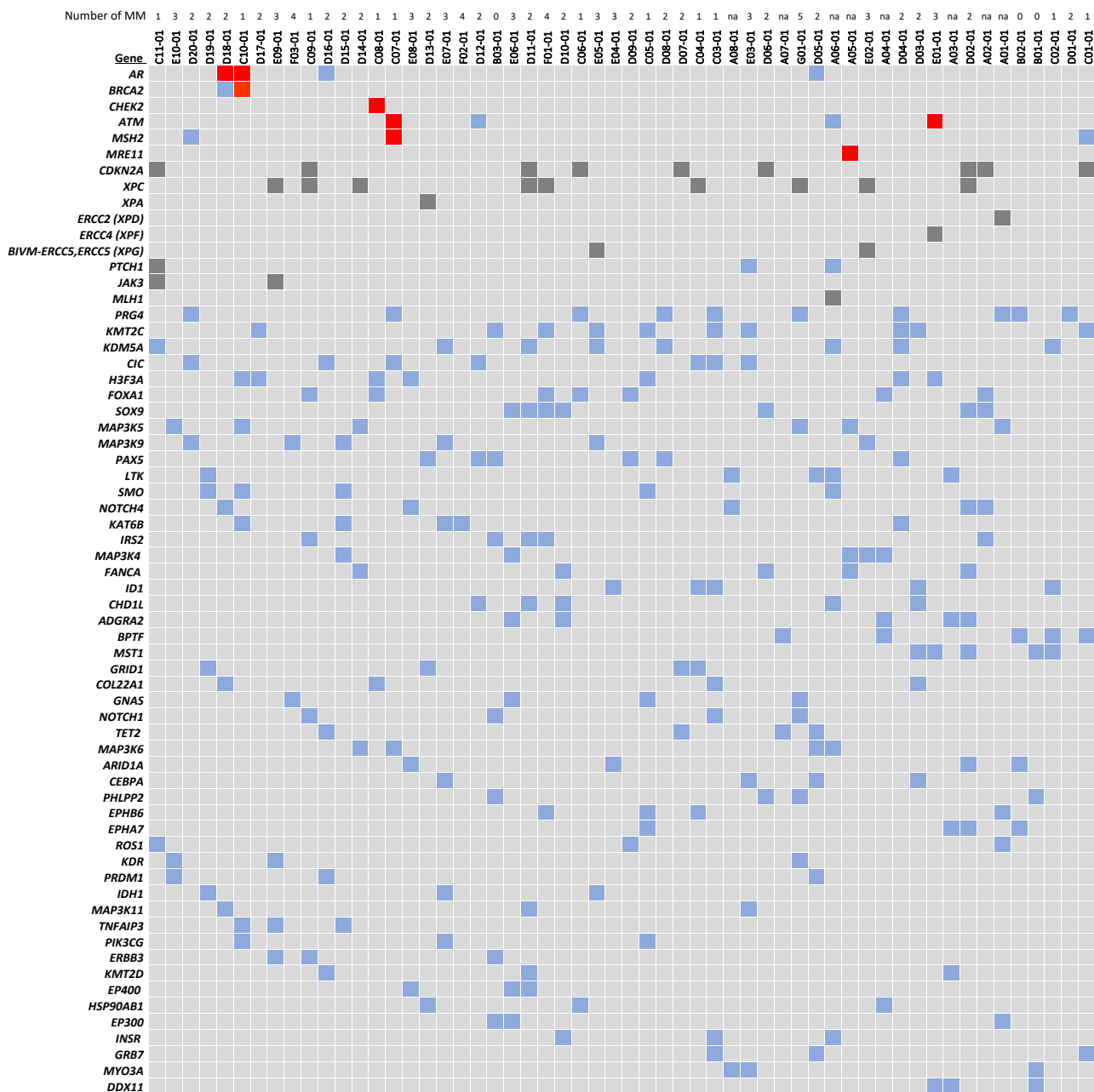

Supplement: Supplementary file 2 — Fig. S2. Germline variants in melanoma‐prone families. Extended oncoplot presenting all germline variants detected in index patients of melanoma‐prone families. Red squares indicate pathogenic or likely pathogenic variants, blue squares indicate variants of uncertain significance (VUS) while dark gray squares indicate variants previously defined as benign but with a possible enrichment in the present cohort. Numbers on top indicate the number of primary malignant melanomas in each index individual. [file MOL2-19-1493-s002.pdf]

Family D18

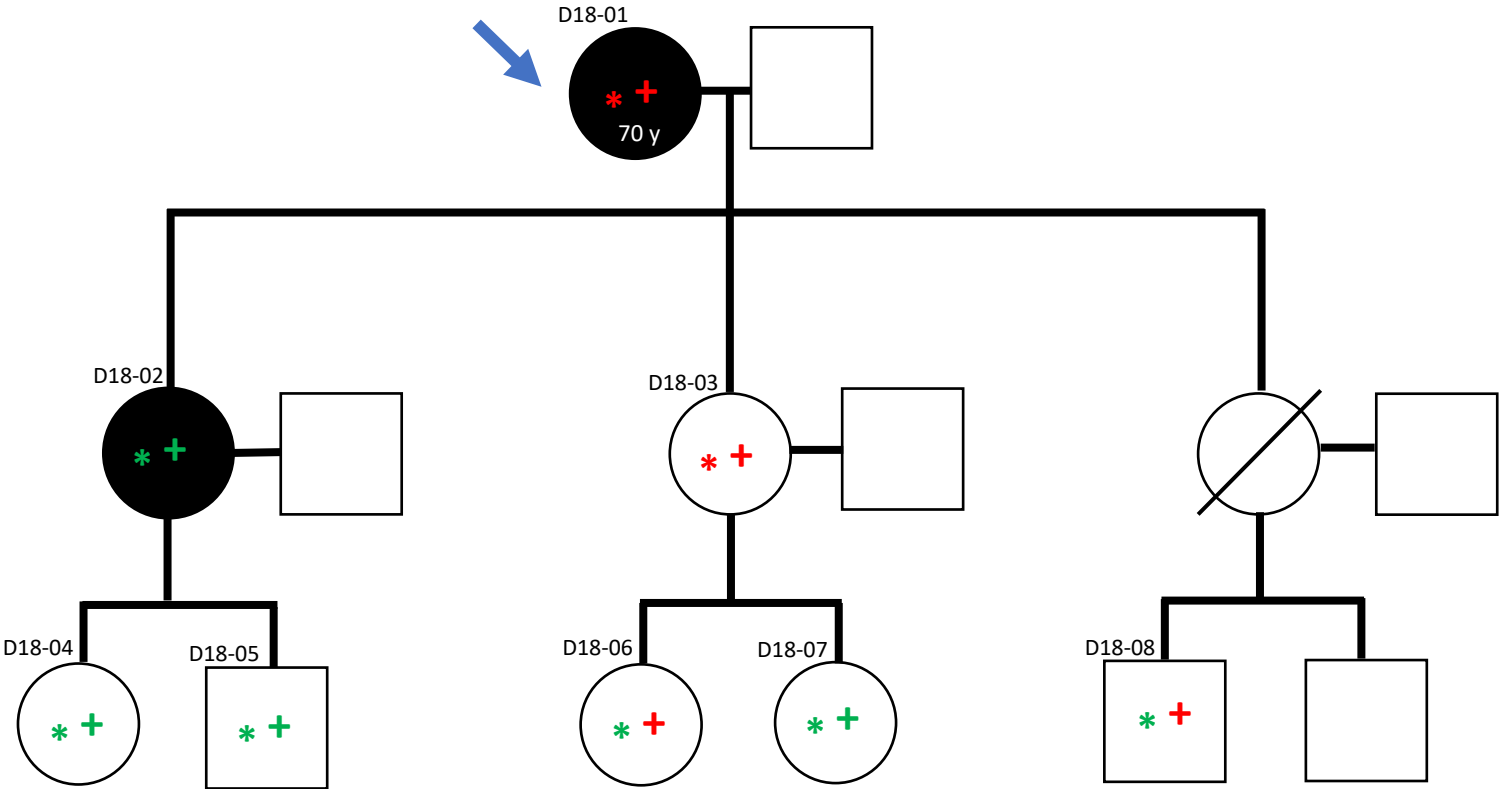

Supplement: Supplementary file 3 — Fig. S3. Family pedigree for family D18. Blue arrow indicates the index individual. Asterisk (*) indicates individual status for the BRCA2:NM_000059:exon4:c.A341G:p.H114R variant; red asterisk indicates variant carriers while green asterisk indicates wild‐type allele only. A plus symbol (+) indicates individual status for the AR:NM_000044:exon6:c.C2395G:p.Q799E variant; red + indicates variant carriers while green + indicates wild‐type allele only. Black color indicates a diagnosis of malignant melanoma while white color indicates no melanoma diagnosis. Diagonal line indicates deceased individuals. [file MOL2-19-1493-s007.pdf]

Supplementary figure S4

Family D14

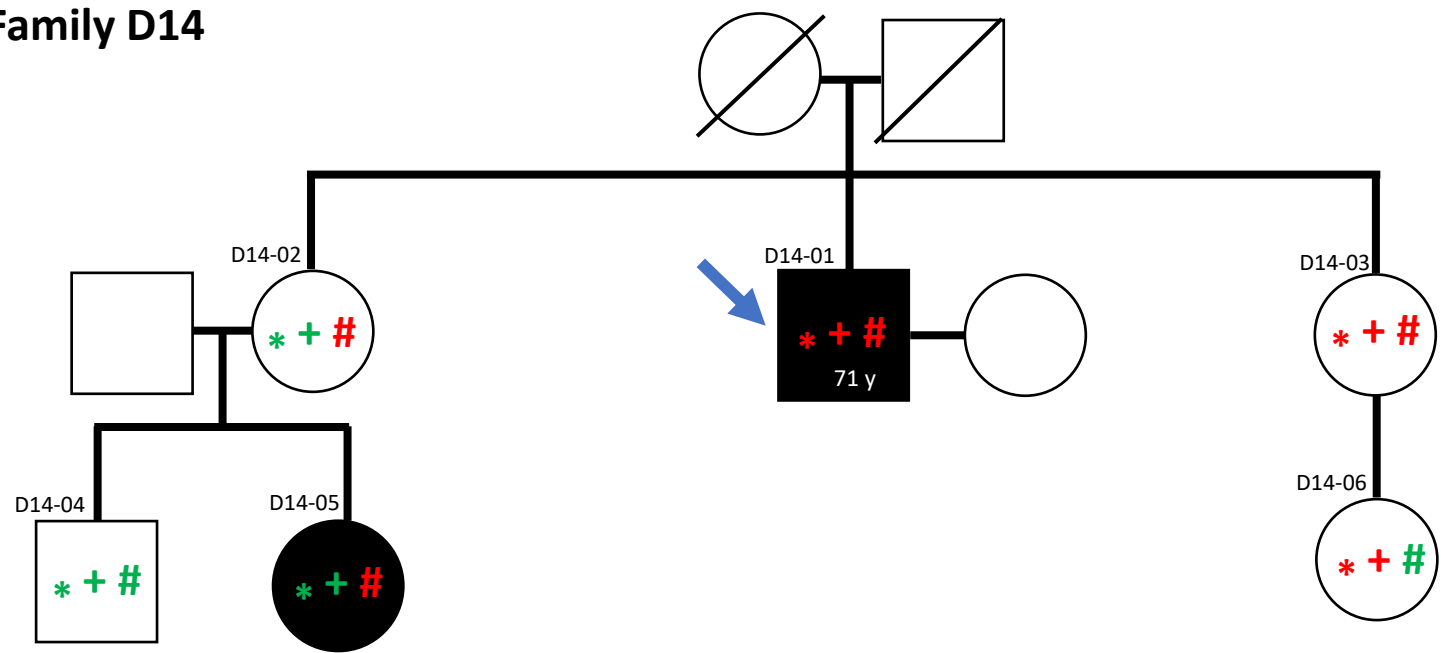

Supplement: Supplementary file 4 — Fig. S4. Family pedigree for family D14. Blue arrow indicates the index individual. Asterisk (*) indicates individual status for the ERBB4:NM_005235:exon12:c.G1451A:p.R484K variant; red asterisk indicates variant carriers while green asterisk indicates wild‐type allele only. A plus symbol (+) indicates individual status for the XPC:NM_004628:exon2:c.C142T:p.L48F variant; red + indicates variant carriers while green + indicates wild‐type allele only. A hash symbol (#) indicates individual status for the NF1:NM_000267:exon5:c.T528A:p.D176E variant; red # indicates variant carriers while green # indicates wild‐type allele only. Black color indicates a diagnosis of malignant melanoma while white color indicates no melanoma diagnosis. Diagonal line indicates deceased individuals. [file MOL2-19-1493-s009.pdf]

Family A06

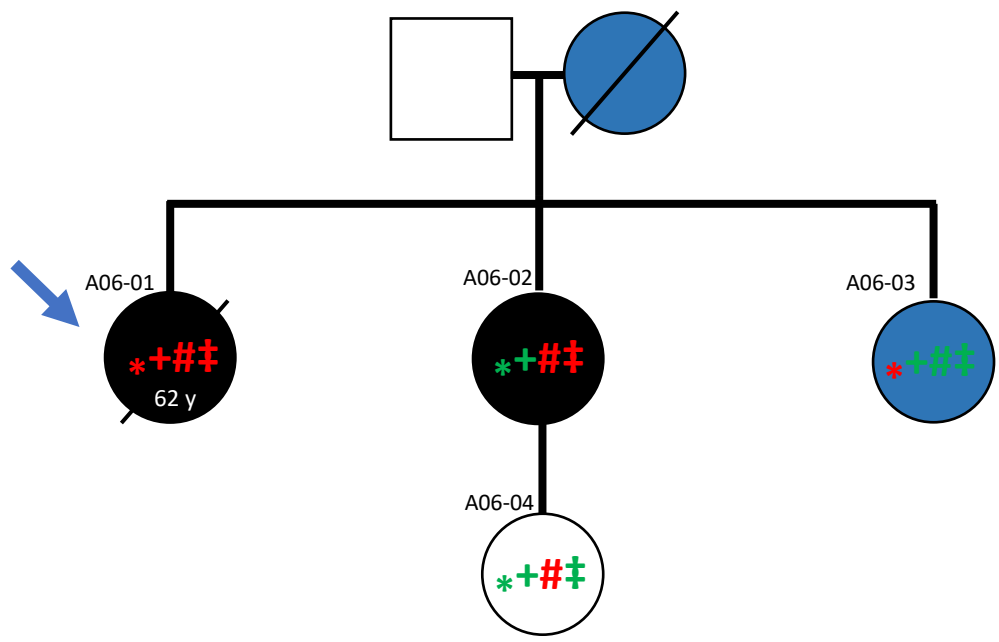

Supplement: Supplementary file 5 — Fig. S5. Family pedigree for family A06. Blue arrow indicates the index individual. Asterisk (*) indicates individual status for the MLH1: NM_000249:exon12:c.G1321A:p.A441 variant; red asterisk indicates variant carriers while green asterisk indicates wild‐type allele only. A plus symbol (+) indicates individual status for a second MLH1 variant, NM_000249:exon16:c.AA1852‐1853GC: p.K618A variant; red + indicates variant carriers while green + indicates wild‐type allele only. A hash symbol (#) indicates individual status for the PTCH1: NM_000264: exon14:c.CA2215TT:p.H739F variant; red # indicates variant carriers while green # indicates wild‐type allele only. A double cross (‡) indicates individual status for the PALB2:NM_024675:exon8:c.T2816G:p.L939W variant; red indicates variant carriers while green indicates wild‐type allele only. Black color indicates a diagnosis of malignant melanoma while white color indicates no melanoma diagnosis. Blue color indicates cancer diagnosis other than melanoma; here stomach cancer (mother of index) and uterine cancer (sister of index). [file MOL2-19-1493-s008.pdf]

# Supplementary figure S6

## Family C11

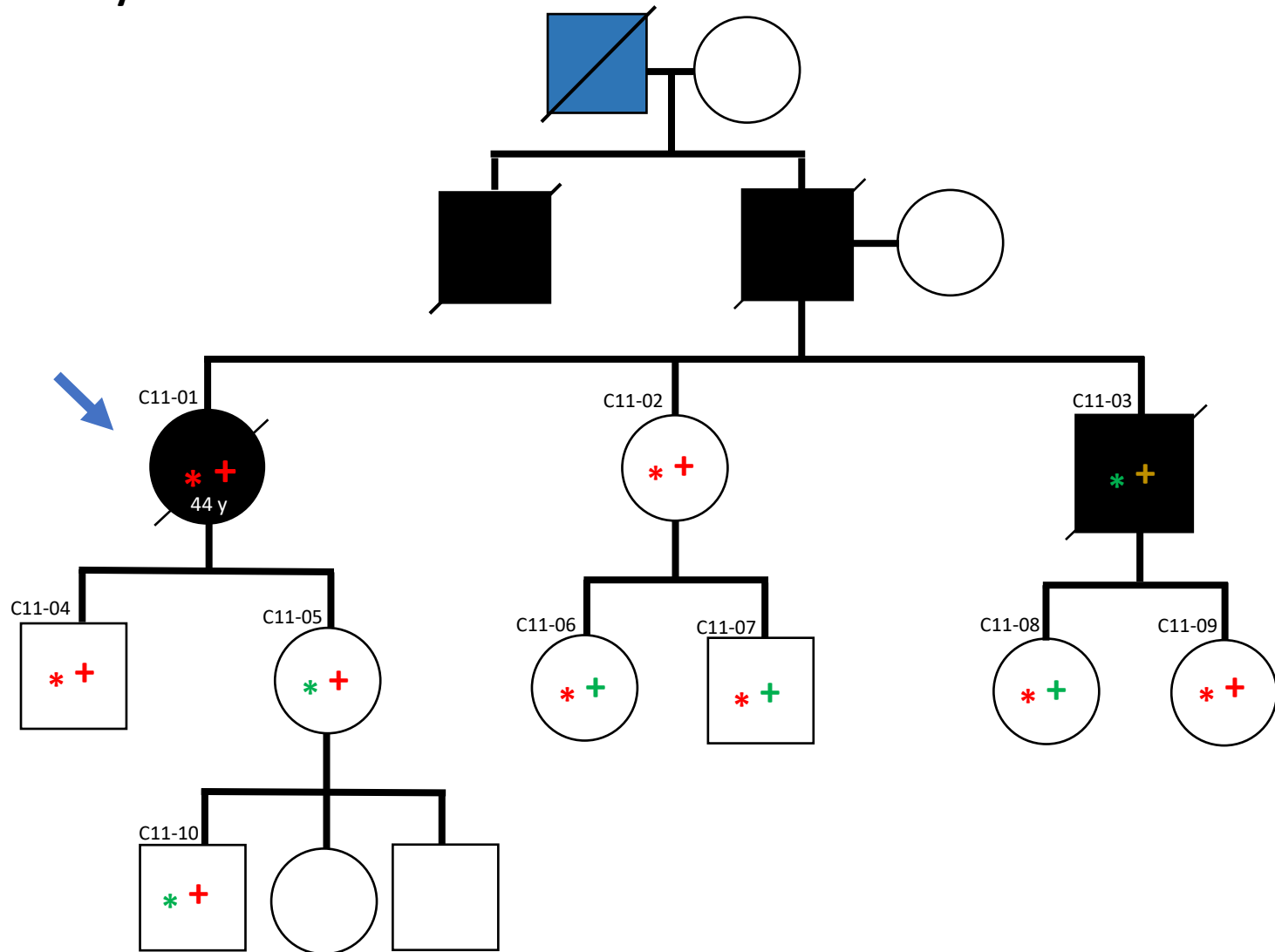

Supplement: Supplementary file 6 — Fig. S6. Family pedigree for family C11. Blue arrow indicates the index individual. Asterisk (*) indicates individual status for the PTCH1:NM_000264:exon14:c.G1994A:p.R665H variant; red asterisk indicates variant carriers while green asterisk indicates wild‐type allele only. A plus symbol (+) indicates individual status for the JAK3:NM_000215:exon16:c.G2164A:p.V722I variant; red + indicates variant carriers while green + indicates wild‐type allele only. Black color indicates a diagnosis of malignant melanoma while white color indicates no melanoma diagnosis. Blue color indicates cancer diagnosis other than melanoma (brain tumor). Diagonal line indicates deceased individuals. Yellow plus symbol indicates that the variant status is unknown due to restricted biomaterial and consequently, technical failure in analysis. [file MOL2-19-1493-s001.pdf]

Supplementary figure S7

Family E09

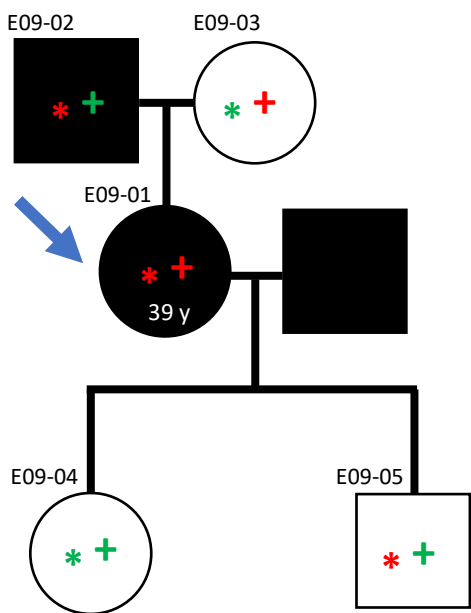

Supplement: Supplementary file 7 — Fig. S7. Family pedigree for family E09. Blue arrow indicates the index individual. Asterisk (*) indicates individual status for the XPC:NM_004628:exon2:c.C142T:p.L48F variant; red asterisk indicates variant carriers while green asterisk indicates wild‐type allele only. A plus symbol (+) indicates individual status for the JAK3:NM_000215:exon16:c.G2164A:p.V722I variant; red + indicates variant carriers while green + indicates wild‐type allele only. Black color indicates a diagnosis of malignant melanoma while white color indicates no melanoma diagnosis. [file MOL2-19-1493-s005.pdf]
