# Supplementary material for: Germline variants in CDKN2A wild‐type melanoma prone families
Source: Mol Oncol. 2025 Mar 12;19(5):1493–507. doi: 10.1002/1878-0261.70020 (PMC12077288; doi:10.1002/1878-0261.70020)
Supplement: Supplementary file 8 — Table S1. Sequencing metrics. Table S2. Gene sequencing panel. Table S3. Primers and annealing temperatures for PCRs. Table S4. Allele frequencies of variants in XP‐related genes. Table S5. Allele frequencies of selected MC1R variants. Table S6. Allele frequencies of all MC1R variants. [file MOL2-19-1493-s003.docx]

**Supplementary Tables**

**Supplementary Table S1**. Sequencing metrics

| Sample ID | Total number of sequenced reads  “TOTAL_READS” ^b^ | Total number of uniquely mapped non-duplicate reads^a^  “PF_UQ_READS_ALIGNED” ^b^ | Total number of covered targeted bases  “ON_BAIT_BASES “^b^ | Median coverage (and range) per targeted base  “MEDIAN_TARGET_COVERAGE” (MIN-MAX) ^b^ | The fraction of targets that did not reach coverage=1 over any base. (Given as %)    “ZERO_CVG_TARGETS_PCT” ^b^ | Percentage of targeted bases with coverage ≥100 (Given as %)  “PCT_TARGET_BASES_100X” ^b^ |
| --- | --- | --- | --- | --- | --- | --- |
| C11-01 | 7405966 | 7099479 | 331388909 | 224 (0-1215) | 0.3056 | 92.83 |
| E10-01 | 7156706 | 6946787 | 253332672 | 172 (0-1288) | 0.3056 | 85.14 |
| D20-01 | 8274744 | 7969344 | 353984313 | 238 (0-1521) | 0.3056 | 94.21 |
| D19-01 | 7291834 | 6947981 | 313613557 | 209 (0-1539) | 0.3056 | 90.57 |
| D18-01 | 8978096 | 8592974 | 389121301 | 260 (0-1811) | 0.3056 | 95.09 |
| C10-01 | 7907026 | 7620310 | 335367592 | 226 (0-1614) | 0.2697 | 93.34 |
| D17-01 | 7394860 | 7117861 | 326689007 | 220 (0-1350) | 0.2697 | 92.87 |
| E09-01 | 7774412 | 7484424 | 325911461 | 219 (0-1387) | 0.3236 | 92.89 |
| F03-01 | 8612092 | 8189956 | 208242747 | 140 (0-857) | 0.2877 | 75.95 |
| C09-01 | 6450366 | 6130746 | 305980947 | 199 (0-1555) | 0.2877 | 87.86 |
| D16-01 | 7086328 | 6650409 | 292762946 | 195 (0-1677) | 0.3056 | 90.64 |
| D15-01 | 7312450 | 6840022 | 309115874 | 205 (0-1676) | 0.2877 | 90.58 |
| D14-01 | 9249314 | 8821853 | 175371447 | 119 (0-675) | 0.2337 | 64.54 |
| C08-01 | 8729844 | 8204230 | 438201189 | 284 (0-1606) | 0.3056 | 94.22 |
| C07-01 | 9143576 | 8694841 | 260930947 | 175 (0-1246) | 0.2877 | 87.52 |
| E08-01 | 8020740 | 7647259 | 351227268 | 231 (0-1780) | 0.3236 | 91.62 |
| D13-01 | 7728230 | 7425876 | 350152091 | 232 (0-1575) | 0.3056 | 91.46 |
| E07-01 | 7897734 | 7602161 | 332865770 | 219 (0-1574) | 0.2877 | 91.61 |
| F02-01 | 6781902 | 6494080 | 323352643 | 213 (0-1514) | 0.3416 | 89.89 |
| D12-01 | 6494740 | 6236044 | 301551208 | 199 (0-1415) | 0.3416 | 88.42 |
| B03-01 | 8301124 | 7910623 | 392928599 | 260 (0-1916) | 0.3056 | 94.07 |
| E06-01 | 7046766 | 6742326 | 341454117 | 224 (0-1433) | 0.3056 | 90.45 |
| D11-01 | 7152408 | 6863364 | 55563314 | 220 (0-1484) | 0.2877 | 90.21 |
| F01-01 | 7217056 | 6936318 | 326218638 | 215 (0-1400) | 0.2517 | 90.41 |
| D10-01 | 7383834 | 7039648 | 357588398 | 233 (0-1715) | 0.2877 | 90.35 |
| C06-01 | 5166246 | 4971573 | 210241794 | 139 (0-836) | 0.3056 | 75.67 |
| E05-01 | 6581388 | 6322637 | 269394468 | 177 (0-1171) | 0.2877 | 86.77 |
| E04-01 | 5749226 | 5592676 | 177318184 | 118 (0-767) | 0.2877 | 63.97 |
| D09-01 | 6603524 | 6295855 | 285911843 | 189 (0-968) | 0.2697 | 89.09 |
| C05-01 | 8135002 | 7772684 | 329141695 | 218 (0-1286) | 0.3056 | 92.33 |
| D08-01 | 7009590 | 6751874 | 261707705 | 175 (0-1249) | 0.2877 | 87.96 |
| D07-01 | 6766818 | 6468089 | 298562632 | 198 (0-1457) | 0.2877 | 89.96 |
| C04-01 | 7130028 | 6828337 | 318821134 | 212 (0-1380) | 0.3056 | 92.90 |
| C03-01 | 7587270 | 6909475 | 348133799 | 222 (0-1099) | 0.3236 | 92.44 |
| A08-01 | 6538752 | 6020449 | 285831561 | 182 (0-1232) | 0.2877 | 88.31 |
| E03-01 | 7391784 | 6798307 | 322603294 | 207 (0-1168) | 0.3236 | 92.84 |
| D06-01 | 8126882 | 7403655 | 350819455 | 223 (0-1425) | 0.3236 | 92.79 |
| A07-01 | 8719468 | 7945674 | 394115975 | 251 (0-1758) | 0.2697 | 94.44 |
| G01-01 | 8158870 | 7569711 | 335693963 | 217 (0-1538) | 0.2517 | 92.24 |
| D05-01 | 7300742 | 6771864 | 333076070 | 217 (0-1431) | 0.3416 | 92.19 |
| A06-01 | 8161768 | 7574408 | 364675984 | 236 (0-1603) | 0.3236 | 94.69 |
| A05-01 | 8122304 | 7722766 | 362982290 | 191 (0-1093) | 0.2854 | 94.18 |
| E02-01 | 7890916 | 7163246 | 370262043 | 190 (0-1059) | 0.2854 | 93.14 |
| A04-01 | 7471074 | 6802390 | 340057195 | 176 (0-920) | 0.2675 | 93.03 |
| D04-01 | 9328186 | 8400243 | 437405037 | 223 (0-1603) | 0.321 | 95.23 |
| D03-01 | 8031512 | 7333244 | 377728231 | 193 (0-1200) | 0.2497 | 93.58 |
| E01-01 | 8666474 | 7766810 | 417023378 | 206 (0-1157) | 0.3032 | 94.26 |
| A03-01 | 8467904 | 7849190 | 400666085 | 208 (0-1367) | 0.2854 | 95.32 |
| D02-01 | 6796066 | 6567925 | 317156795 | 195 (0-922) | 0.2497 | 89.39 |
| A02-01 | 6969632 | 6738512 | 325200738 | 167 (0-935) | 0.321 | 91.49 |
| A01-01 | 9197068 | 8725149 | 416164686 | 222 (0-1317) | 0.2854 | 94.85 |
| B02-01 | 8210502 | 7959306 | 358203757 | 194 (0-1007) | 0.3745 | 93.56 |
| B01-01 | 9466396 | 9169262 | 417082225 | 226 (0-1302 | 0.3032 | 94.64 |
| C02-01 | 8037808 | 7830073 | 358680827 | 195 (0-1021) | 0.3032 | 93.49 |
| D01-01 | 8110266 | 7854828 | 368760495 | 201 (0-1013) | 0.321 | 93.89 |
| C01-01 | 8351998 | 8119944 | 374031012 | 203 (0-1192) | 0.2854 | 94.16 |

a: Reference genome used was GRCh37

b: HS-metric field as defined at:

[https://broadinstitute.github.io/picard/picard-metric-definitions.html#HsMetrics](https://broadinstitute.github.io/picard/picard-metric-definitions.html#HsMetrics)

**Supplementary Table S2**. Gene sequencing panel (360 analyzed genes)

| **ABL1** | **CARD11** | **DICER1** | **FGFR3** | **INHBA** | **MAPK10** | **NFE2L2** | **PRKCI** | **SOCS1** |
| --- | --- | --- | --- | --- | --- | --- | --- | --- |
| **ABL2** | **CASP8** | **DNMT3A** | **FGFR4** | **INSR** | **MAPK7** | **NGFR** | **PTCH1** | **SOX10** |
| **ACVR2A** | **CBL** | **DYRK1B** | **FH** | **IRS2** | **MAPK8** | **NKX2-1** | **PTCH2** | **SOX2** |
| **AKT1** | **CCND1** | **E2F3** | **FLT1** | **JAK1** | **MAPK9** | **NOTCH1** | **PTEN** | **SOX9** |
| **AKT2** | **CCND2** | **EEF1A2** | **FLT3** | **JAK2** | **MCL1** | **NOTCH2** | **PTK6** | **SPOP** |
| **AKT3** | **CCND3** | **EGFR** | **FLT4** | **JAK3** | **MDM2** | **NOTCH3** | **PTP4A1** | **SRC** |
| **ALK** | **CCNE1** | **EIF5A2** | **FOXA1** | **JUN** | **MDM4** | **NOTCH4** | **PTP4A3** | **SRSF2** |
| **APC** | **CD79A** | **ELK3** | **FOXL2** | **KAT6B** | **MED12** | **CCN3** | **PTPN11** | **STAT3** |
| **AR** | **CD79B** | **EP300** | **FOXO1** | **KDM5A** | **MED12L** | **NPM1** | **RAB23** | **STK11** |
| **ARAF** | **CDC6** | **EP400** | **FOXP4** | **KDM5C** | **MED13** | **NRAS** | **RAB25** | **SUFU** |
| **ARFRP1** | **CDC73** | **EPHA3** | **GAB2** | **KDM6A** | **MED29** | **NTRK1** | **RAC1** | **TBX22** |
| **ARID1A** | **CDH1** | **EPHA5** | **GABRG1** | **KDR** | **MEN1** | **NTRK2** | **RAF1** | **TBX3** |
| **ARID1B** | **CDH2** | **EPHA6** | **GATA1** | **KEAP1** | **MET** | **NTRK3** | **RARA** | **TERT** |
| **ARID2** | **CDH20** | **EPHA7** | **GATA2** | **KIT** | **MITF** | **OR5L1** | **RB1** | **TET2** |
| **ASXL1** | **CDH5** | **EPHB1** | **GATA3** | **KRAS** | **MLH1** | **PAK1** | **REG4** | **TGFBR2** |
| **ATM** | **CDK12** | **EPHB4** | **GATA6** | **LGALS7** | **KMT2A** | **PAK3** | **RET** | **TMPRSS2** |
| **ATR** | **CDK4** | **EPHB6** | **GNA11** | **LTK** | **KMT2D** | **PALB2** | **RICTOR** | **TOP1** |
| **ATRX** | **CDK6** | **ERBB2** | **GNAQ** | **MAFA** | **KMT2C** | **PARP10** | **RNF43** | **TP53** |
| **AURKA** | **CDK8** | **ERBB3** | **GNAS** | **MAP2K1** | **MPL** | **PAX5** | **ROBO1** | **TP63** |
| **AURKB** | **CDKN1B** | **ERBB4** | **GPC5** | **MAP2K2** | **MRAS** | **PAX9** | **ROBO2** | **TP73** |
| **AXIN1** | **CDKN2A** | **ERCC2** | **ADGRA2** | **MAP2K4** | **MRE11** | **PBRM1** | **ROS1** | **TRAF2** |
| **BAG4** | **CDKN2B** | **ERCC3** | **GRB2** | **MAP2K5** | **MSH2** | **PDGFRA** | **RPS6KB1** | **TSC1** |
| **BAP1** | **CDKN2C** | **ERCC4** | **GRB7** | **MAP2K6** | **MSH6** | **PDGFRB** | **RPTOR** | **TSC2** |
| **BCL11A** | **CEBPA** | **ERCC5** | **GRID1** | **MAP2K7** | **MST1** | **PHF6** | **RRM2B** | **TSHR** |
| **BCL2** | **CHD1** | **ESR1** | **GUCY1A2** | **MAP3K1** | **MTDH** | **PHGDH** | **RSPO2** | **U2AF1** |
| **BCL2A1** | **CHD1L** | **ETV1** | **H3-3A** | **MAP3K11** | **MTOR** | **PHLPP2** | **RSPO3** | **USP9X** |
| **BCL2L1** | **CHEK1** | **EZH2** | **H3C2** | **MAP3K12** | **MUTYH** | **PIK3C3** | **RUNX1** | **VEGFA** |
| **BCL2L2** | **CHEK2** | **FADD** | **HMGA2** | **MAP3K13** | **MYB** | **PIK3CA** | **SETD2** | **VHL** |
| **BCL6** | **CIC** | **AMER1** | **HNF1A** | **MAP3K14** | **MYC** | **PIK3CG** | **SF3B1** | **NSD3** |
| **BCOR** | **CKS1B** | **FANCA** | **HOXA3** | **MAP3K2** | **MYCL** | **PIK3R1** | **SFTPA1** | **WSB1** |
| **BIRC2** | **COL22A1** | **FANCC** | **HRAS** | **MAP3K3** | **MYCN** | **PIK3R2** | **SHC1** | **WT1** |
| **BIRC7** | **CREBBP** | **FANCD2** | **HSP90AB1** | **MAP3K4** | **MYD88** | **PLCG1** | **SKP2** | **XPA** |
| **BLM** | **CRKL** | **FANCE** | **HSP90AA1** | **MAP3K5** | **MYO3A** | **POU1F1** | **SLIT2** | **XPC** |
| **BPTF** | **CTCF** | **FANCF** | **ID1** | **MAP3K6** | **MYO5B** | **PPM1D** | **SMAD2** | **XPO1** |
| **BRAF** | **CTNNB1** | **FANCG** | **IDH1** | **MAP3K7** | **MYOC** | **PPP2R1A** | **SMAD3** | **YAP1** |
| **BRCA1** | **DAXX** | **FAS** | **IDH2** | **MAP3K8** | **NBN** | **PRDM1** | **SMAD4** | **YWHAB** |
| **BRCA2** | **DCUN1D1** | **FBXO11** | **IGF1R** | **MAP3K9** | **NCOA2** | **PRDM9** | **SMARCA4** | **YWHAQ** |
| **BRIP1** | **DDR2** | **FBXW7** | **IGF2R** | **MAP4K3** | **NCOA3** | **PREX2** | **SMARCB1** | **YWHAZ** |
| **BUB1B** | **DDX11** | **FGFR1** | **IKBKE** | **MAP4K4** | **NF1** | **PRG4** | **SMO** | **ZNF217** |
| **EMSY** | **DDX3X** | **FGFR2** | **IL7R** | **MAPK1** | **NF2** | **PRKCG** | **SMURF1** | **ZRSR2** |

**Supplementary Table S3.** Primers and annealing temperatures for PCRs.

| **Gene** | **Variant** | **Forward primer** | **Sequence** | **Tm °C** | **Family** |
| --- | --- | --- | --- | --- | --- |
| CDKN2A | 13kbp deletion | CDKN2A Del-For | AGCTCATCCCAGTTCAAGACC | 56,6 | all indexes |
|  |  | CDKN2A Del-Rev | CAGCTCCTCAGCCAGGTCC |  |  |
| PTCH1 | NM_000264: exon14:c.G1994A:p.R665H | PTCH1-ex14-For | ACACCGACACACACGACAATAC | 58 | C11 |
|  |  | PTCH1-ex14-Rev | CCTTGTGGAGCTGGTGCTCTCTG |  |  |
| JAK3 | NM_000215:exon16:c.G2164A:p.V722I | JAK3-ex15-For1 | GAGGCGCAGACACTTAGCTTG | 64,9 | C11, E09 |
|  |  | JAK3-ex15-Rev3 | GCACTGTTGAATCAGCAG |  |  |
| BRCA2 | NM_000059:exon4:c.A341G:p.H114 | BRCA2-ex4-For | TCACTGAATTATTGTACTG | 54 | D18 |
|  |  | BRCA2-ex4-Rev | GATCTTCTACCAGGCTCT |  |  |
| BRCA2 | NM_000059: exon11:c.G5857T:p.E1953X | BRCA2-ex11-For | ACGAGGCATTGGATGATTCAGAG | 68 | C10 |
|  |  | BRCA2-ex11-Rev | AATGAAGCATCTGATACCTGGACA |  |  |
| NF1 | NM_000267:exon5:c.T528A:p.D176E | NF1-ex5-For | ACCTGTCCCCTAATACTTAA | 59,6 | D14 |
|  |  | NF1-ex5-Rev2 | GGTGTTCTAGTTCAGCACA |  |  |
| ATM | NM_000051:exon13:c.C1931A:p.S644X | ATM-ex14-For | CTTTCTTGAAGTGAACACCACC | 58 | C07 |
|  |  | ATM- ex14-Rev | GCGATCCAGTGATTCCTTGAGA |  |  |
| MLH1 | NM_000249:exon12:c.G1321A:p.A441T | MLH1-ex12-For | AGGTCTATGCCCACCAGATG | 66,1 | A06 |
|  |  | MLH1-ex12-Rev | ATTCCAGCACCATTCCAGAG |  |  |
| MLH1 | NM_000249:exon16:c.AA1852GC:p.K618A | MLH1-ex16-For2 | AGGCACTTCCTTTGGGGTAT | 58 |  |
|  |  | MLH1-ex16-Rev | CTCCCAAAGTGCTGGGATTA |  |  |
| PTCH1 | NM_000264: exon14: c.AC2216TT: p.H739Y | PTCH1-ex14CA-For | GTGACACAGGACACCCTCAG | 66,1 |  |
|  |  | PTCH1-ex14CA-Rev | CACTCCCATGGAAGATGACC |  |  |
| PALB2 | NM_024675:exon8:c.T2816G:p.L939W | PALB2-ex8-For | TCTGAGCCTTCAAATGATGAAA | 58 |  |
|  |  | PALB2-ex8-Rev2 | CTAGGTTATTACCTGCACT |  |  |
| MRE11 | NM_005590:exon4:c.201delC:p.S68Qfs*12 | MRE11-ex4-For2 | TGGTGTAATTGGAAGATGGCT | 63,5 | A05 |
|  |  | MRE11-ex4-Rev2 | TGGAAGGCAAAACAGTTGTG |  |  |
| MAP3K6 | NM_004672: exon19: c.2544delC: p.F849Sfs*143 | MAP3K6_ex19_For | CCCCAGAAATCATTGACCAG | 62,1 |  |
|  |  | MAP3K6_ex19_Rev | GGGTGAACAAGGGTGACATT |  |  |
| ATM | NM_000051:exon22:c.3244_3247insTGdelC:p.H1082Lfs*13 | ATM-ex23-For | TGCTTGAGGTGAGTTTTTGC | 60,5 | E01 |
|  |  | ATM-ex23-Rev | TGTAAGACATTCTACTGCCATCTG |  |  |
| ERCC4/  XPF | NM_005236:exon11:c.A2546T:p.Q849L | ERCC4-ex11-For | GGAGCTGAAACAAAGCAAGC | 60,5 | E01 |
|  |  | ERCC4-ex11-Rev | AGGAGCCGCTGAAAAGTACA |  |  |
| XPC | NM_004628:exon2:c.C142T:p.L48F | XPC-ex2-For | GGAGACAGGTCATAGAGCCG | 63 | E09, C09, D14,  D11, F01, C04,  E02, D02 |
|  |  | XPC-ex2-Rev2 | CACCTGAGGTCATCCCCATC |  |  |
| XPA | NM_000380:exon1:c.G16A:p.G6R | XPA exon1-For | ATGACAAGAGAGCAGGTAG | 59,2 | D13 |
|  |  | XPA exon1-Rev | CGCACCGAGGCAGGCAGC |  |  |
| MC1R | This gene is only one exon, primers were designed to cover it. | MC1R For1 | TGGCAGCACCATGAACTAAG | 63,5 | all indexes |
|  |  | MC1R Rev1 | ATGAAGAGCGTGCTGAAGAC |  |  |
|  |  | MC1R For2 | CATCTCCATCTTCTACGC | 63,5 |  |
|  |  | MC1R Rev2 | CCACACAATATCACCACCT |  |  |

**Supplementary Table S4.** Allele frequencies of variants in XP-related genes.

| **Variant** | **Cohort^1^** | **Ref alleles** | **Alt alleles** | **Allele freq.** | **p-value^2^** |
| --- | --- | --- | --- | --- | --- |
|  |  |  |  |  |  |
| *XPA* G6R | iFM | 111 | 1 | 0.009 |  |
|  | 1K genomes (eur) | 1006 | 0 | 0 | 0.100 |
|  | gnomAD (Sw.) | 25612 | 16 | 0.001 | 0.071 |
|  |  |  |  |  |  |
| *XPC* L48F | iFM | 104 | 8 | 0.071 |  |
|  | 1K genomes (eur) | 984 | 22 | 0.022 | 0.007 |
|  | gnomAD (Sw.) | 22551 | 647 | 0.028 | 0.014 |
|  |  |  |  |  |  |
| *XPC* K481N | iFM | 111 | 1 | 0.009 |  |
|  | 1K genomes (eur) | 996 | 10 | 0.010 | 1.000 |
|  | gnomAD (Sw.) | 25265 | 143 | 0.006 | 0.470 |
|  |  |  |  |  |  |
| *ERCC2* P426L | iFM | 111 | 1 | 0.009 |  |
|  | 1K genomes (eur) | 1006 | 0 | 0 | 0.100 |
|  | gnomAD (Sw.) | 26130 | 0 | 0 | 0.004 |
|  |  |  |  |  |  |
| *ERCC4* Q849L | iFM | 111 | 1 | 0.009 |  |
|  | 1K genomes (eur) | 1006 | 0 | 0 | 0.100 |
|  | gnomAD (Sw.) | 26126 | 4 | 1.5E-04 | 0.021 |
|  |  |  |  |  |  |
| *ERCC5* E399K | iFM | 111 | 1 | 0.009 |  |
|  | 1K genomes (eur) | 1006 | 0 | 0 | 0.100 |
|  | gnomAD (Sw.) | 26132 | 0 | 0 | 0.004 |
|  |  |  |  |  |  |
| *ERCC5* A1036T | iFM | 111 | 1 | 0.009 |  |
|  | 1K genomes (eur) | 1006 | 0 | 0 | 0.100 |
|  | gnomAD (Sw.) | 26092 | 42 | 0.002 | 0.168 |
|  |  |  |  |  |  |
|  |  |  |  |  |  |
| Total^3^ | iFM | 98 | 14 | 0.125 |  |
|  | 1K genomes (eur) | 974 | 32 | 0.032 | 7.1E-05 |
|  |  |  |  |  |  |

^1^ iFM: index individuals of the present study, 1K genomes (eur): European population of the 1000 genomes study version aug2015-phase 3, gnomAD (Sw.): Swedish population in gnomAD, version 2.1.1.

^2^ Calculated against FM (the present study).

^3^ Accurate calculations not possible for gnomAD, due to varying total number of individuals analyzed for each variant.

**Supplementary Table S5.** Allele frequencies of selected *MC1R* variants.

| **Variant** | **Cohort^1^** | **Ref alleles** | **Alt alleles** | **Allele freq.** | **p-value^2^** |
| --- | --- | --- | --- | --- | --- |
|  |  |  |  |  |  |
| D84E | iFM | 108 | 4 | 0.036 |  |
|  | 1K genomes (eur) | 996 | 10 | 0.010 | 0.043 |
|  | gnomAD (Sw.) | 25667 | 227 | 0.009 | 0.018 |
|  |  |  |  |  |  |
| D294H | iFM | 109 | 3 | 0.027 |  |
|  | 1K genomes (eur) | 998 | 8 | 0.008 | 0.089 |
|  | gnomAD (Sw.) | 25690 | 214 | 0.008 | 0.067 |
|  |  |  |  |  |  |
| N29fs | iFM | 109 | 3 | 0.027 |  |
|  | 1K genomes (eur) | 1003 | 3 | 0.003 | 0.016 |
|  | gnomAD (Sw.) | 25173 | 133 | 0.005 | 0.022 |
|  |  |  |  |  |  |
| A111V | iFM | 111 | 1 | 0.009 |  |
|  | 1K genomes (eur) | 1006 | 0 | 0 | 0.100 |
|  | gnomAD (Sw.) | 25850 | 10 | 3.9E-04 | 0.046 |
|  |  |  |  |  |  |
| C315R | iFM | 111 | 1 | 0.009 |  |
|  | 1K genomes (eur) | 1006 | 0 | 0 | 0.100 |
|  | gnomAD (Sw.) | 25865 | 1 | 3.9E-05 | 0.009 |
|  |  |  |  |  |  |
|  |  |  |  |  |  |
| Total^3^ | iFM | 100 | 12 | 0.107 |  |
|  | 1K genomes (eur) | 985 | 21 | 0.021 | 3.3E-05 |
|  |  |  |  |  |  |

^1^ iFM: index individuals of the present study, 1K genomes (eur): European population of the 1000 genomes study version aug2015-phase 3, gnomAD (Sw.): Swedish population in gnomAD, version 2.1.1.

^2^ Calculated against FM (the present study).

^3^ Accurate calculations not possible for gnomAD, due to varying total number of individuals analyzed for each variant.

**Supplementary Table S6.** Allele frequencies of all *MC1R* variants.

| **Variant** | **Cohort^1^** | **Ref alleles** | **Alt alleles** | **Allele freq.** | **p-value^2^** |
| --- | --- | --- | --- | --- | --- |
|  |  |  |  |  |  |
| D84E | iFM | 108 | 4 | 0.036 |  |
|  | 1K genomes (eur) | 996 | 10 | 0.010 | 0.043 |
|  | gnomAD (Sw.) | 25667 | 227 | 0.009 | 0.018 |
|  |  |  |  |  |  |
| D294H | iFM | 109 | 3 | 0.027 |  |
|  | 1K genomes (eur) | 998 | 8 | 0.008 | 0.089 |
|  | gnomAD (Sw.) | 25690 | 214 | 0.008 | 0.067 |
|  |  |  |  |  |  |
| N29fs | iFM | 109 | 3 | 0.027 |  |
|  | 1K genomes (eur) | 1003 | 3 | 0.003 | 0.016 |
|  | gnomAD (Sw.) | 25173 | 133 | 0.005 | 0.022 |
|  |  |  |  |  |  |
| A111V | iFM | 111 | 1 | 0.009 |  |
|  | 1K genomes (eur) | 1006 | 0 | 0 | 0.100 |
|  | gnomAD (Sw.) | 25860 | 10 | 3.9E-04 | 0.046 |
|  |  |  |  |  |  |
| C315R | iFM | 111 | 1 | 0.009 |  |
|  | 1K genomes (eur) | 1006 | 0 | 0 | 0.100 |
|  | gnomAD (Sw.) | 25865 | 1 | 3.9E-05 | 0.009 |
|  |  |  |  |  |  |
| R151C | iFM | 93 | 19 | 0.170 |  |
|  | 1K genomes (eur) | 934 | 72 | 0.072 | 0.001 |
|  | gnomAD (Sw.) | 23901 | 1985 | 0.077 | 0.001 |
|  |  |  |  |  |  |
| R160W | iFM | 98 | 14 | 0.125 |  |
|  | 1K genomes (eur) | 944 | 62 | 0.062 | 0.017 |
|  | gnomAD (Sw.) | 23076 | 2744 | 0.106 | 0.537 |
|  |  |  |  |  |  |
| V60L | iFM | 102 | 10 | 0.089 |  |
|  | 1K genomes (eur) | 893 | 113 | 0.112 | 0.527 |
|  | gnomAD (Sw.) | 23379 | 2511 | 0.097 | 1.000 |
|  |  |  |  |  |  |
| V92M | iFM | 103 | 9 | 0.080 |  |
|  | 1K genomes (eur) | 937 | 69 | 0.069 | 0.694 |
|  | gnomAD (Sw.) | 23853 | 2015 | 0.078 | 0.860 |
|  |  |  |  |  |  |
| R163Q | iFM | 106 | 6 | 0.054 |  |
|  | 1K genomes (eur) | 936 | 70 | 0.070 | 0.692 |
|  | gnomAD (Sw.) | 23986 | 1832 | 0.071 | 0.582 |
|  |  |  |  |  |  |
|  |  |  |  |  |  |
| Total^3^ | iFM | 42 | 70 | 0.625 |  |
|  | 1K genomes (eur) | 599 | 407 | 0.405 | 1.2E-05 |
|  |  |  |  |  |  |

^1^ iFM: index individuals of the present study, 1K genomes (eur): European population of the 1000 genomes study version aug2015-phase 3, gnomAD (Sw.): Swedish population in gnomAD, version 2.1.1.

^2^ Calculated against FM (the present study).

^3^ Accurate calculations not possible for gnomAD, due to varying total number of individuals analyzed for each variant.
