# Supplementary material for: Germline variants in CDKN2A wild‐type melanoma prone families
Source: Mol Oncol. 2025 Mar 12;19(5):1493–507. doi: 10.1002/1878-0261.70020 (PMC12077288; doi:10.1002/1878-0261.70020)
Supplement: Supplementary file 9 — Data S1. Materials and methods. [file MOL2-19-1493-s006.docx]

**Supplementary Materials and Methods**

**Patients**

For the present study we included 56 index individuals. These were either previously treated for multiple primary melanomas, or for a single melanoma but with several relatives with a melanoma diagnosis. Index individuals without a melanoma diagnosis themselves but with several relatives with melanoma were also allowed. All types of primary melanomas were allowed (cutaneous melanoma, uveal melanomas, melanomas of the genitourinary tract etc.). We also included patients with metastatic melanoma of unknow origin when other family members had been diagnosed with melanoma as well. All patients were at least 18 years old and gave their written informed consent prior to participation. Relatives of all enrolled patients were invited to participate as well, if aged >18 years. All in all, 273 relatives were enrolled in the trial following extensive genetic counselling and written informed consent. Most index individuals had previously tested negative for pathogenic germline variants in *CDKN2A* in routine diagnostics but were centrally reanalysed after inclusion in the present study. In a few cases, for validation of inheritance when blood samples were not available, we were able to recover melanoma tumor tissue samples from deceased relatives, known to be diagnosed with a melanoma during their lifetime.

**DNA isolation from blood samples**

DNA from full blood was isolated using the QIAamp DNA Mini Kit (Qiagen, Hilden, Germany*)* according to the *DNA Purification from Blood or Body Fluids* spin protocol provided by the manufacturer, with the exception that 400µl fullblood was used as starting material. Elution was performed twice with 100µl AE buffer, yielding a total volume of 200µl purified DNA.

**DNA isolation from Saliva samples**

Individuals that were not able to deliver blood samples (study ID C06-06, C06-03 and A05-40) donated saliva samples instead. DNA was isolated from saliva using the Oragene DNA OG-600 kit *(DNA genotek, Ottawa, Canada)*. The procedure was performed according to manufacturer’s protocol for purification from 0,5ml sample. In brief, the sample collection tube was incubated at 50°C in hot-air incubator for three hours, 0,5ml sample were transferred to a new tube and mixed with 20µl PT-L2P reagent. Samples were incubated on ice for 10 minutes followed by centrifugation of turbidity for 5 minutes at 15000g. The clear supernatant was mixed with 600µl absolute ethanol in a new tube and incubated 10 minutes for DNA precipitation. The DNA were pelleted by centrifugation for 2 minutes at 15000g followed by ethanol wash before dissolving the DNA pellet in 50µl 10mM Tris-HCl (0.5mM EDTA, pH 9). Finally, for total rehydration, the DNA were left over night in elution buffer at room temperature.

**Extraction of DNA from FFPE samples**

In several families, relatives of the index patients were deceased when the present study was conducted, and blood or saliva samples of these relatives were therefore not available. In some cases, formalin fixated paraffin embedded (FFPE) tumour specimens from these individuals were available. Tissue was collected from FFPE blocks either as sections of, on average, 20µm thickness, or as cores of 1mm size. DNA was isolated as previously described (8). In brief, the Covaris adaptive focused acoustics (AFA) procedure was used, according to manufactures protocol, section C, with minor changes, applying a Covaris M220 focused-ultrasonicator (Covaris). Elution volume was 50µl BE buffer (5mM Tris HCl, pH 8.5). Prior to further analyses, the isolated DNA were repaired from damages induced by the FFPE procedure, using the PreCR repair mix (New England Biolabs) and dNTP mixture (Takara) according to manufactures sequential reaction protocol.

**Multiplex Ligand Probe Amplification (MLPA)**

Small copy number variation in index patients was analysed with Multiplex ligand probe amplification (MLPA) assay and Coffalyser software (v. 140721.1958 and v. 220401.0000). SALSA MLPA Probe kit P419-A2, specific for *CDKN2A*, *CDKN2B* and *CDK4* was used (MRC Holland Amsterdam, The Netherlands) and the assay was performed according to the manufacturer’s manual. As positive control, we included DNA from a previous study, where a deletion was known to remove the MLPA probe binding site in exon 1α (9).

***CDKN2A* 13kb deletion screening**

All index cases were screened for a 13 kb deletion in *CDKN2A*, previously detected in a Norwegian melanoma prone family (9). Analyses were performed, by a PCR specific for the deletion’s breakpoint, as previously described (9) (Supplementary Fig. S1).

**Targeted cancer gene panel sequencing**

Massive parallel DNA sequencing of a targeted 360-cancer gene panel was carried out as previously described (10). As starting material, gDNA isolated from fullblood was used for all index patients. For some relatives, blood was not available and FFPE blocks were used as starting material for DNA isolation, as described above. In brief, Illumina libraries were prepared applying Kapa Hyper Prep kit (Kapa Biosystem) and Agilent SureSelect XT-kit (Agilent). Targeted enrichment was performed using RNA baits (SureSelect, Agilent), targeted against an in-house panel of 360 cancer related genes, +/- 5bp of each exon to cover splice sites (10) . Libraries were sequenced on a MiSeq instrument (Illumina) to an average mean depth of 231x for blood samples and 171x for FFPE samples.

**PCR amplification and Sanger Sequencing**

Regions with variants of interest, detected in index cases, were analysed in family members by PCR amplification and capillary sequencing. In addition, the *MC1R* gene was not covered by our 360 gene panel and therefore analysed separately by PCR and capillary sequencing. The specific regions were amplified with specific primers and Taq Polymerase (VWR) according to manufactures instructions. In general, amplification was carried out in the final concentrations in a mastermix of PCR-clean water, 1x Key buffer with MgCl, 0,2mM dNTPs, 0,2µM forward and reverse primer (Supplementary Table S1) and Taq polymerase. 1µl gDNA was used as template, for low quality DNA from FFPE 3-5µl was used. PCR thermocycling started with denaturation at 95 ˚C for five minutes followed by 35 cycling of 95 ˚C for 30 seconds, TM ˚C (see Supplementary Table S1) for 30 seconds and 72 ˚C 30 seconds. A final elongation at 72 ˚C for 10 minutes was followed by cooling the samples to 10 ˚C. Correct size of the amplified product was confirmed by agarose gel electrophoresis along with Generuler DNA ladder mix (Thermo Scientific). Amplified PCR products were cleaned up prior sequencing reaction with Exozap Illustra Exoprostar 1-step enzymatic PCR and sequencing reaction clean-up kit (GE Healthcare life Sciences). Amplification for capillary sequencing was conducted according to manufacturer’s recommendations with BigDye terminator version 1.1 (Applied Biosystems) and appropriate primers in a 10µl reaction volume. Thermocycling were denaturation at 94˚C for 5 minutes followed by 30 cycles of 94˚C for 15 seconds, 50˚C for 5 seconds and 60˚C for 4 minutes. Finally, the samples were cooled to 10˚C. Capillary chromatograms were obtained by analyses on an automated DNA sequencer ABI 3730 DNA Analyzer.

**Data analysis of Miseq sequencing**

Targeted 360 cancer-gene panel sequencing data were first analysed (mapped to the human reference genome hg19 and variant calling) with the built-in Miseq Reporter software on the Miseq instrument (Illumina) before the variant output files (vcf files) were annotated with ANNOVAR for gene context (11). Post-processing filters were applied to variant output data, resulting in a list of variants sharing the same properties. These were only exonic variants with variant allele frequency of more than 0.1, while synonymous single nucleotide variants with less than 5 mutated reads and depth lower than 20x were excluded. All variants were subjected to population filtering using the data from 1000GenomesProject_2014okt_all, 1000GenomesProject_2014okt_ EUR and Esp6500siv2_all, to keep the variants with less than 3% minor allele frequency (MAF). All variants of interest were validated by manual inspection of mapping and read quality of sequence reads in IGV. In the main analysis, the resulting variants were grouped as either pathogenic, variant of uncertain significance (VUS) or benign based on literature review and assessment of information in publicly available databases, including Clinvar, Cosmic, PubMed, the Genome Aggregation Database and databases specific for some genes of interest, such as BRCA Exchange. In a secondary, more stringent analysis, we performed pathogenicity assessment of variants, applying the Charger algorithm (12) as previously described (13).

**Ethics**

The study protocol was approved by the institutional review board, i.e. the Regional Ethics Committee for medical research in the South-Eastern Health Region of Norway (REK sør-øst 2015/941). All participants provided written informed consent to genetic testing. All individuals with pathogenic or suspected pathogenic variants were referred to genetic counselling. Ethical approval was received from the regional ethics committee (REK sør-øst 27863-2020) to recover archival tumor tissue / biopsy material from deceased relatives in a few families, if necessary, to strengthen the genetic findings of our study.
